# A Configurable Event-Driven Convolutional Node with Rate Saturation Mechanism for Modular ConvNet Systems Implementation

**DOI:** 10.3389/fnins.2018.00063

**Published:** 2018-02-20

**Authors:** Luis A. Camuñas-Mesa, Yaisel L. Domínguez-Cordero, Alejandro Linares-Barranco, Teresa Serrano-Gotarredona, Bernabé Linares-Barranco

**Affiliations:** ^1^Instituto de Microelectrónica de Sevilla (IMSE-CNM), CSIC y Universidad de Sevilla, Sevilla, Spain; ^2^Department of Computer Architectures, University of Sevilla, Sevilla, Spain

**Keywords:** convolutional neural networks, neuromorphic vision, Address Event Representation (AER), event-driven processing, neural network hardware, Reconfigurable Networks

## Abstract

Convolutional Neural Networks (ConvNets) are a particular type of neural network often used for many applications like image recognition, video analysis or natural language processing. They are inspired by the human brain, following a specific organization of the connectivity pattern between layers of neurons known as receptive field. These networks have been traditionally implemented in software, but they are becoming more computationally expensive as they scale up, having limitations for real-time processing of high-speed stimuli. On the other hand, hardware implementations show difficulties to be used for different applications, due to their reduced flexibility. In this paper, we propose a fully configurable event-driven convolutional node with rate saturation mechanism that can be used to implement arbitrary ConvNets on FPGAs. This node includes a convolutional processing unit and a routing element which allows to build large 2D arrays where any multilayer structure can be implemented. The rate saturation mechanism emulates the refractory behavior in biological neurons, guaranteeing a minimum separation in time between consecutive events. A 4-layer ConvNet with 22 convolutional nodes trained for poker card symbol recognition has been implemented in a Spartan6 FPGA. This network has been tested with a stimulus where 40 poker cards were observed by a Dynamic Vision Sensor (DVS) in 1 s time. Different slow-down factors were applied to characterize the behavior of the system for high speed processing. For slow stimulus play-back, a 96% recognition rate is obtained with a power consumption of 0.85 mW. At maximum play-back speed, a traffic control mechanism downsamples the input stimulus, obtaining a recognition rate above 63% when less than 20% of the input events are processed, demonstrating the robustness of the network.

## 1. Introduction

The concept of neuromorphic engineering was first proposed by Carver Mead back in the 1980s based on the analogy between the behavior of transistors biased in sub-threshold region and the physics in biological neurons (Mead, 1989). This approach opened a new processing paradigm which takes inspiration from the structure and operation of the human brain (Sterling and Laughlin, 2015): information encoded in spikes (also called events) which are processed in parallel by massive layers of neurons interconnected via synapses.

In recent years, the development of bio-inspired event-driven neuromorphic Dynamic Vision Sensors (DVS) (Lichtsteiner et al., 2008; Posch, 2011; Serrano-Gotarredona and Linares-Barranco, 2013) provides a new and revolutionary way of capturing visual scenes by the generation of flows of events accurately representing the motion of real objects. In a DVS, each pixel operates autonomously and sends an output event (spike) whenever it senses a change of light greater than a preset threshold. This way, a continuous flow of events with a high temporal resolution (sub-microsecond) is obtained, representing moving reality as it changes, without waiting to assemble or scan artificial time-constrained frames. These flows of events can be processed by Spiking Neural Networks (SNNs), performing complex tasks like object tracking (Delbrück and Lang, 2013) or shape recognition (Zhao et al., 2015).

A particular type of SNNs are the event-driven Convolutional Neural Networks (ConvNets), where the interconnections between layers of neurons do not follow an all-to-all pattern. In a ConvNet, each neuron from layer *i* is connected only to a subset of neurons in layer *i*+1, known as projective field. These projective fields can be represented by a 2D convolutional kernel, and imply an important reduction in the amount of synapse memory in a network, which facilitates its hardware implementation. These Convolutional Neural Networks were originally developed for frame-driven processing (LeCun et al., 1989), training them with static images, although some methods have been proposed to transform a frame-driven ConvNet into an event-driven one implemented in software (Pérez-Carrasco et al., 2013; Diehl et al., 2015), and other methods directly train the event-driven networks with spikes(Orchard et al., 2015). In the present work, we propose a hardware implementation of event-driven ConvNets that can process visual information from a DVS in real time, avoiding time-consuming software approaches.

The current evolution of hardware neuromorphic platforms tends to large-scale modular computing systems with increasing numbers of neurons and synapses (Indiveri et al., 2011; Liu et al., 2015; Furber, 2016). Some successful approaches are the IBM TrueNorth (Merolla et al., 2014), the Stanford Neurogrid (Benjamin et al., 2014), the Heidelberg BrainScaleS (Schemmel et al., 2010) and the Manchester SpiNNaker (Furber et al., 2014). These projects used different techniques to design hierarchically scalable networks with multiple chips per board, and multiple boards per rack, assembling systems with between 1 and 460 millions of neurons and between 1 and 460 billions of synapses, obtaining power consumptions between 100 mW and 50*kW* (Furber, 2016).

In a previous work, we developed an event-driven convolutional unit with 64 × 64 neurons in VLSI which could be used to build larger arrays and implement arbitrary ConvNets (Camuñas-Mesa et al., 2012). However, this approach presented two important limitations: the excessive physical size of a complex network, and the lack of a saturation mechanism in the I&F neurons, which is necessary for a multi-layer ConvNet.^1^ Zamarreño-Ramos et al. (2013) proposed a scalable approach based on reconfigurable networks implemented on FPGA to overcome the first limitation, presenting up to 262 k neurons and 32 millions of synapses, including routing capabilities in the convolutional nodes. (Pérez-Carrasco et al., 2013) proposed an exact method to map the saturation from a conventional frame-based description to an event-driven system, and tested it in software. Recently, rectifying non-saturating non-linearities like ReLUs (Rectified Linear Units) have been proposed as an alternative to rate saturation mechanism in frame-based systems (Cao et al., 2015; Diehl et al., 2015). However, ReLUs are not a good solution for spiking hardware implementations, because if a neuron in a layer becomes excessively active it will generate a large amount of spikes and can collapse the communication network.

In this work, we designed a configurable convolutional unit for FPGAs that can be used to build large-scale ConvNets, including a programmable rate saturation mechanism that reproduces the refractory period of biological neurons, allowing to transform conventional frame-based networks into equivalent event-driven implementations. This unit has been tested and characterized for rate saturation period values between 50μs and 51.2 ms. This unit has been designed to assemble large 2D arrays (Zamarreño-Ramos et al., 2013), and a whole ConvNet with 22 convolutional blocks trained for poker card symbol recognition has been implemented in one Spartan6 FPGA. This network included 5 k neurons and 500 k synapses within a single FPGA. More complex hierarchical structures using larger FPGAs, and assembling multiple FPGAs in a PCB and multiple PCBs in a rack, can potentially be used to implement very large-scale Convolutional Neural Networks. While other neuromorphic approaches are based on expensive dedicated hardware, the proposed architecture allows for implementing arbitrary ConvNets on cheap commercial FPGAs. The implemented network was tested with a stimulus where 40 poker cards are observed by a DVS in 1 s time window. Different slow-down factors were applied, from real time processing to 100 times slower, obtaining recognition rates as high as 96% with a power consumption of 0.85 mW.

The paper is organized as follows. Section 2.1 describes the convolutional node in detail, with special emphasis on the rate saturation mechanism, while Section 2.2 details the complete ConvNet implemented for poker card symbol recognition. Section 3 presents the experimental results obtained for characterization of both the individual module and the ConvNet, and, finally, these results are discussed in Section 4.

## 2. Material and methods

This Section describes the proposed configurable convolutional node in Section 2.1 (including details of its main operations in sections 2.1.1, 2.1.2, 2.1.3, and 2.1.4) and the ConvNet implemented for recognition tasks in Section 2.2.

### 2.1. Configurable convolutional node

**Figure 1:**
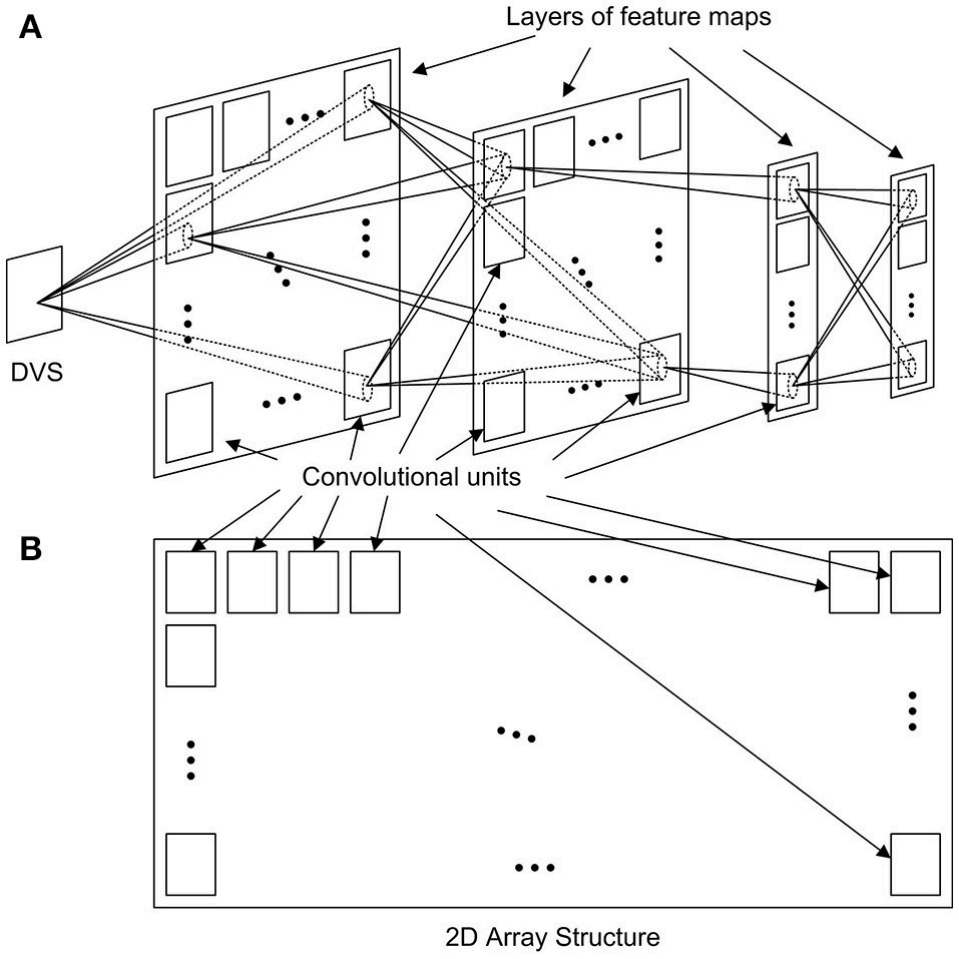
Implementation of **(A)** a generic ConvNet with several layers of feature maps using **(B)** a 2D array structure of convolutional units.

A generic event-driven Convolutional Neural Network (ConvNet) follows the structure represented in Figure 1A, with multiple layers of feature maps, including several convolutional units in each layer. Each unit is formed by a bi-dimensional array of neurons, and receives events from every unit in the previous layer, applying different convolutional kernels depending on the origin of the event, implementing a multi-kernel operation. The aim of this work is to design a 2D array structure in hardware formed by convolutional units which can efficiently implement an arbitrary ConvNet, as illustrated in Figure 1B. For that, a configurable network node is designed.

**Figure 2:**
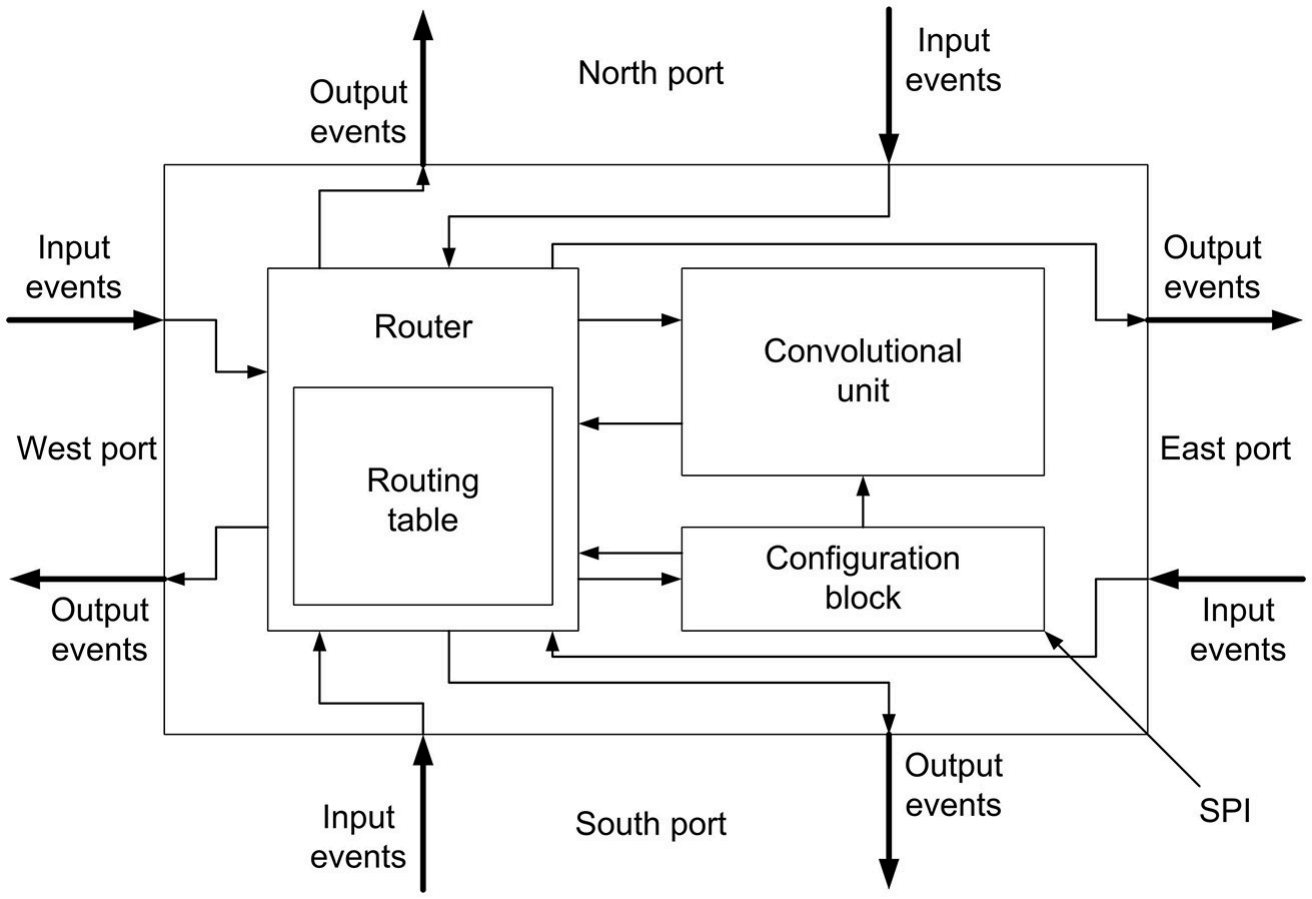
Block diagram for the node designed to build 2D arrays.

The block diagram of the network node is shown in Figure 2, including a convolutional unit formed by a bi-dimensional array (with configurable size) of I&F neurons, a router and a configuration block. This node has been designed to assemble large 2D arrays, where each node is directly connected to 4 other neighboring nodes through North, South, East and West ports. Each of these ports carries bidirectional flows of events (input and output). Internally, all input and output ports are connected to a router, which sends each incoming event to either the appropriate output port or the local convolutional unit, depending on both the event header and the routing table, according to the destination-driven protocol (Zamarreño-Ramos et al., 2013). An internal configuration block receives commands through a Serial Peripheral Interface (SPI) connection, and sends them to the router or to the convolutional unit.

**Figure 3:**
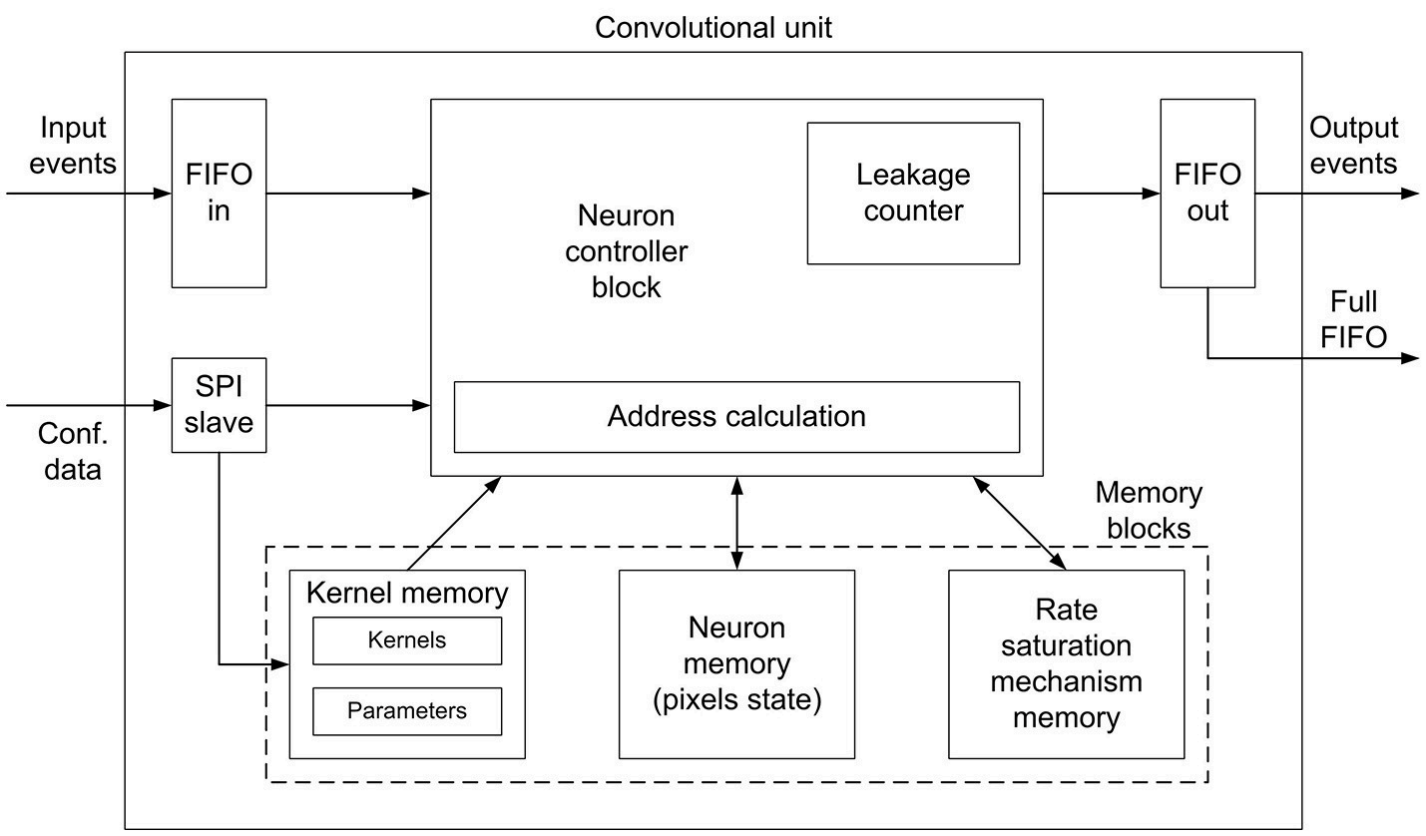
Block diagram for the convolutional unit. A controller block receives input events from an input FIFO, reads the pixels state and the convolutional kernel from the corresponding memory blocks, and sends output events to an output FIFO. An SPI block receives configuration data. A specific memory block is used to implement the rate saturation mechanism. A *full*_*FIFO*_ signal is generated by the output FIFO to implement the traffic control mechanism.

The convolutional unit designed in this work is fully configurable, so that it can be used to implement different nodes (each one with different properties) within complex multi-layer networks. Figure 3 shows the details of the convolutional unit. It computes the convolution of the input events *eν*_*in*_(*t, x, y, p, k*) with a kernel *w*_*k*_(*x, y*), generating output events *eν*_*out*_(*t, x, y, p*), where *t* is time, *x* and *y* are the spatial coordinates, *p* is the polarity of the event, and *k* is the kernel id, as multi-kernel processing is allowed. Input events are stored in an input FIFO, while the controller block reads events from this input FIFO, processes them using integrate and fire neurons (pixels), and writes output events in an output FIFO, which sends them out to the next module. A *full*_*FIFO*_ signal is generated when the output FIFO is full in order to stop receiving more input events, allowing the implementation of a flow control mechanism, which is described later. The state of the convolution (the values of all pixels or neurons) is stored in the Neuron Memory, while the Kernel Memory stores all the kernel values and their corresponding parameters: x- and y-size, and center shift, as shown in **Figure 5B**. If the center shift is zero, the kernel will be applied to a neighborhood of pixels where the one given by the address of the input event is in the middle. A different value of this parameter shifts the position of the kernel before being applied to the pixels. Another memory is used to implement the refractory period mechanism which is described later. The convolutional unit receives configuration data through an SPI interface, which is used to write the kernels (with their parameters) and the parameters of the neuron controller (threshold, leakage, rate saturation period).

The configurability of the convolutional unit includes some parameters which have to be adjusted before the hardware implementation, and some other parameters which can be modified after implementation using the SPI interface, as they are related to the training of the network.

Pre-implementation parameters are the following:

- Address space of input events: maximum values for *x* and *y* accepted by the convolutional unit ($x_{in}^{max},y_{in}^{max}$). These numbers will define the number of bits for $x_{in}^{i}$ and $y_{in}^{i}$ in the input events.
- Address space of output events, which corresponds to the number of pixels in the 2D array ($x_{out}^{max},y_{out}^{max}$). These numbers will define the number of bits for $x_{out}^{i}$ and $y_{out}^{i}$ in the output events.
- Size of the neuron memory. This parameter is given by *n*_*x*_ × *n*_*y*_ × *n*_*bits*_, where (*n*_*x*_, *n*_*y*_) represent the number of pixels in the array and *n*_*bits*_ the resolution of the register where their state is stored.
- Size of the kernel memory. This memory is divided in 2 different blocks: 1) the block where the kernels weights are stored, whose size is given by the maximum number of kernels *N*_*k*_, and the size of each kernel $x_{k}^{max}\times y_{k}^{max}$; and 2) the block where the kernels parameters are stored, being these parameters the size of each kernel and the center shift. Only the size of this whole memory is specified. The number of kernels *N*_*k*_ will define the number of bits needed for the kernel id *k*^*i*^ in the input events.
- Size of the rate saturation memory. This parameter is given by *n*_*x*_ × *n*_*y*_ × *n*_*bits*__*T*__*R*__, where (*n*_*x*_, *n*_*y*_) represent the number of pixels in the array and *n*_*bits*__*T*__*R*__ the resolution of the register used to implement the rate saturation mechanism, as described later.
- Range of the rate saturation period. This parameter (*b*_*TR*_) selects the range of values available for the rate period *T*_*R*_, establishing $T_{R}^{min}$ and $T_{R}^{max}$. The exact value within this range is specified after implementation using the SPI. The programming of the rate period range is described in section 2.1.3.

Post-implementation parameters are:

- Threshold of the integrate and fire pixels. Although the pixel values in theory should vary between −*Th* and *Th*, in practice we work only with positive numbers, so the negative threshold is set to 0, the positive value is set to 2 × *Th* and the reset value is *Th*.
- Leakage parameters: *T*_*leak*_, which indicates the period of the leakage pulses that are applied to all the pixels, and *N*_*leak*_, which indicates the amplitude of the these pulses.
- Rate saturation period (*T*_*R*_), the exact value within the available range established before implementation.
- Kernel values and parameters: values which are written on the kernel memory.

**Figure 4:**
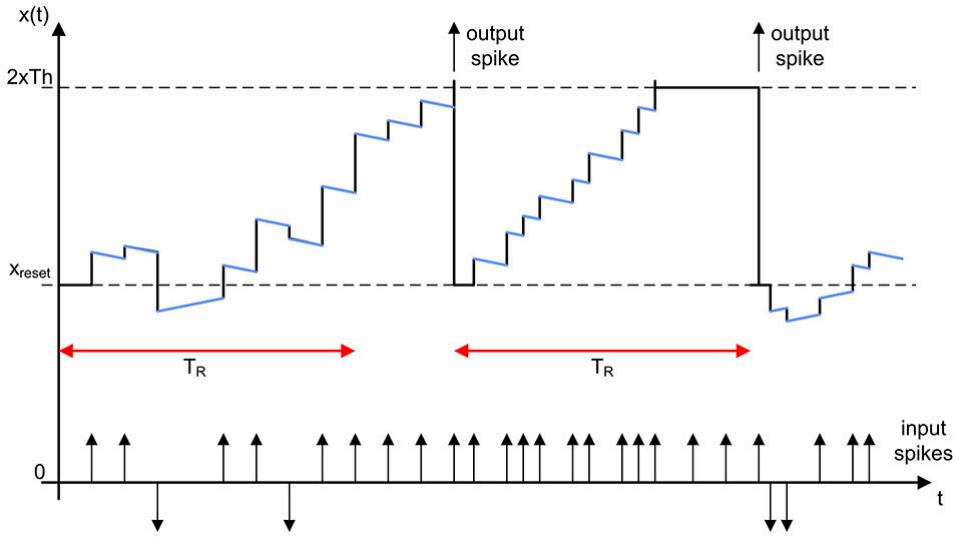
Illustration of the evolution of a neuron while processing input spikes, showing the global leakage effect (represented by blue segments) and the rate saturation period limitation (indicated by red arrows). The state of the neuron is updated with each input spike (increased with positive spikes and decreased with negative ones). If the threshold is reached before *T*_*R*_, the output spike is not generated until the rate saturation time is over.

Figure 4 shows the typical state evolution of an integrate and fire pixel, with reset value *x*_*reset*_ = *Th* and positive threshold 2 × *Th*. The state of the pixel is updated with a positive or negative value each time a new event is processed. The decay of the pixel state represents the global leakage. The rate saturation period *T*_*R*_ imposes a limitation to the minimum time between consecutive output spikes. In this example, the second output spike was not generated when the pixel reached the threshold, but when the rate saturation mechanism allowed for it. The following sections describe in detail the behavior of the convolutional unit.

Concerning the router included in the network node shown in Figure 2, it is based on the 2D structure represented in Figure 1B, where each convolutional unit is identified by its (*x, y*) coordinates. The router receives external events from the four neighboring nodes and, based on its programmed routing table, decides whether to send them to its local convolutional unit or to other neighbor. In particular, we used a destination-driven protocol, so the address of the destination node is written in the routing header of each event, introducing a network layer handled by the routers and transparent to the convolutional units. When the router receives an external event, it reads the addressing header and decides the output port to which the event must be forwarded. If the destination address corresponds to the node address, the event is sent to the local convolutional unit. If this is not the case, it compares the destination address with the present node address to decide the output port to which the event must be forwarded, choosing the shortest path to the destination in terms of number of hops. On the other hand, when the router receives an event from the local convolutional unit, it inserts a header indicating the destination node according to the routing table. When that node is connected to several destination nodes, the router clones the event as many times as the number of destination nodes and writes each address in the corresponding header. Further details about the router are given in (Zamarreño-Ramos et al., 2013).

#### 2.1.1. Convolutional operation

**Figure 5:**
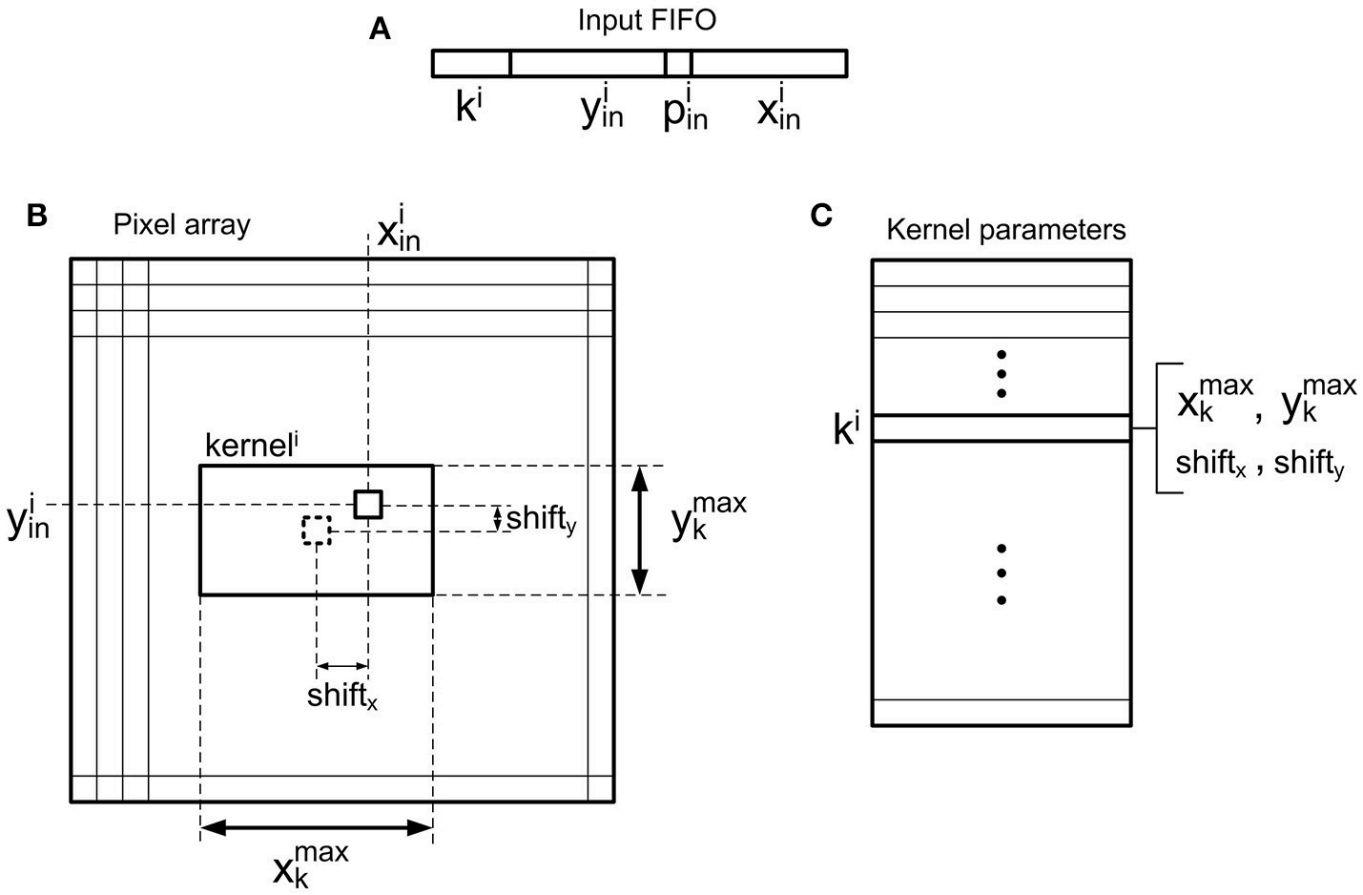
Illustration of the convolutional operation. **(A)** Input event, where $x_{in}^{i}$ and $y_{in}^{i}$ are the spatial coordinates, $p_{in}^{i}$ the polarity (positive or negative), and *k*^*i*^ the kernel id. **(B)** Position of *kernel*^*i*^ inside the pixel array, centered on a pixel given by coordinates $\left(x_{in}^{i}+shift_{x},y_{in}^{i}+shift_{y}\right)$. **(C)** Kernel parameters, where $x_{k}^{max}$, $y_{k}^{max}$ represent the kernel size and *shift*_*x*_, *shift*_*y*_ indicate the position where the convolutional kernel is centered relative to the input event address, as shown in **(B)**.

Every time a new incoming event arrives at the convolutional unit, it is stored in the input FIFO, and as soon as there is at least one event in the FIFO, the controller block performs the following steps:

1. Read the event from the input FIFO, obtaining the address $\left(x_{in}^{i},y_{in}^{i}\right)$, the polarity $p_{in}^{i}$, and the kernel id *k*^*i*^, as shown in Figure 5A.
2. Using the kernel id *k*^*i*^, access the kernel memory (in particular, the parameters block, in Figure 5C) to read the size and center shift of the indicated kernel. This information, together with the event address, gives the coordinates of the pixels which have to be updated with the kernel values, as shown in Figure 5B. If all these coordinates are outside the pixels array, discard the event.
3. Knowing the coordinates of the pixels affected by the incoming event, calculate both the positions of the states of these pixels in the neuron memory, and the positions of the corresponding kernel weights in the kernel memory. This operation is performed by the Address calculation block in Figure 3.
4. One by one, read an individual pixel value (also called partial sum, in CNN terminology) and kernel weight, and calculate the addition of both of them. If the incoming event is negative ($p_{in}^{i}=-1$), invert first the kernel weight.
5. If the result of the addition is larger than the positive threshold (positive event) or smaller than the negative one (negative event), check the minimum period *T*_*R*_ for that specific pixel. If firing event is allowed, go to step 6. If not, update the new pixel value (or partial sum) in the corresponding position of the memory, and wait for the next input event. This missing event will be sent out the first time this pixel receives an input event after *T*_*R*_ is over, introducing an error Δ*t* that will be compensated using the method described in section 2.1.3.
6. Generate an output event with address $\left(x_{out}^{j},y_{out}^{j}\right)$ and polarity $p_{out}^{j}$, and write it in the output FIFO, reset the corresponding pixel and update it in its memory position.

#### 2.1.2. Global leakage

In parallel with the convolution process described before, a global leakage process runs continuously in the controller block. A global 32-bit counter is increased with every clock cycle, until it reaches the previously programmed value *T*_*leak*_. This process has the highest priority, and every time it reaches *T*_*leak*_ it cycles through all the data stored in the Neuron Memory and decreases all neuron values by *N*_*leak*_ if they are positive, and increases them if they are negative, never crossing the reset value. This process makes all neurons converge toward the reset value.

#### 2.1.3. Rate saturation period mechanism

The main novelty of this work is the hardware implementation of a mechanism that emulates the refractory period property of biological neurons. This property guarantees a minimum separation in time (given by *T*_*R*_) between two consecutive spikes generated by a single neuron.

**Figure 6:**
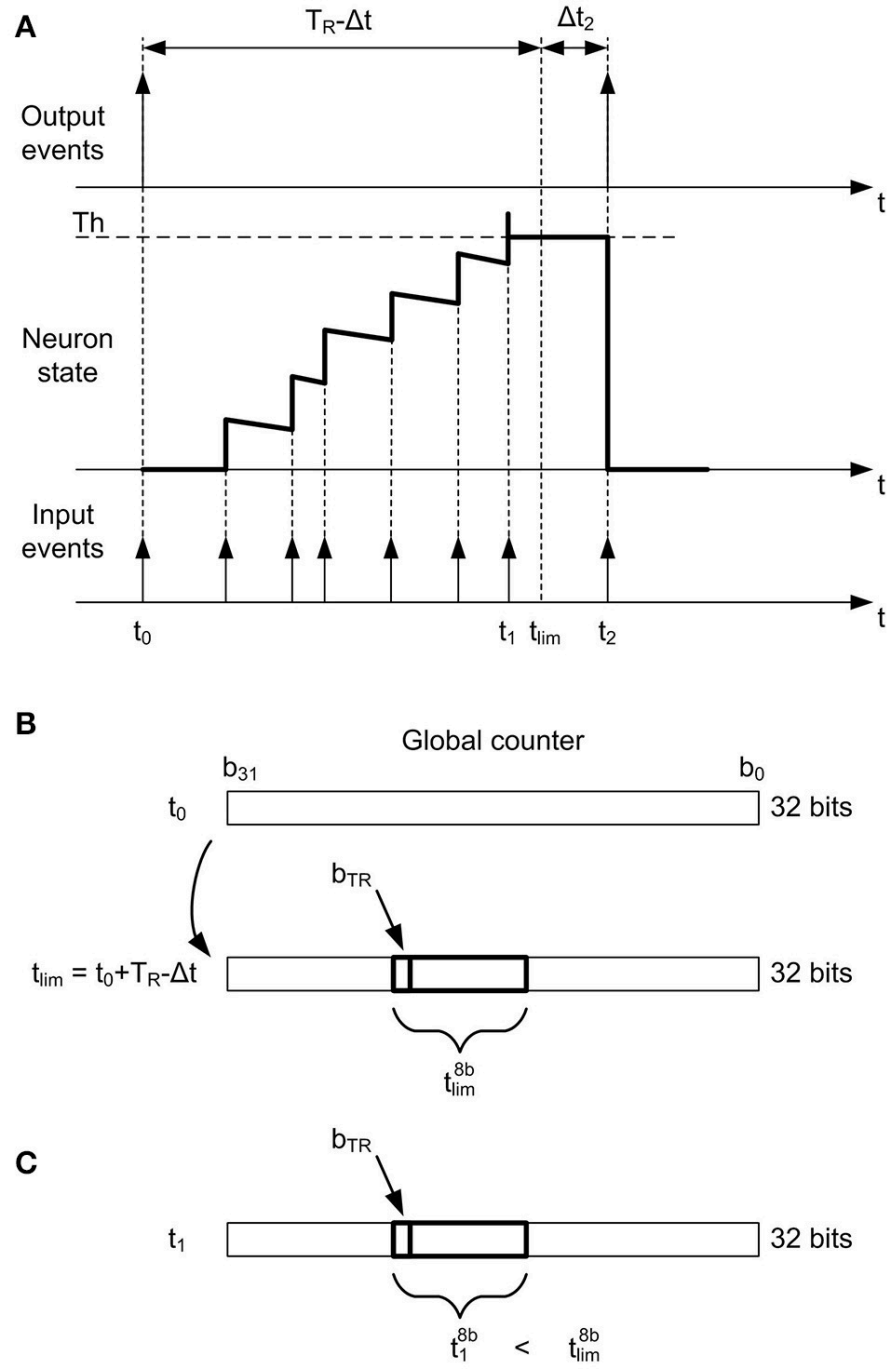
Rate saturation mechanism. **(A)** A pixel generates an output event at *t* = *t*_0_, so no output events will be allowed until *t*_*lim*_ = *t*_0_ + *T*_*R*_ − Δ*t*. The pixel state reaches the threshold at *t*_1_ (<*t*_*lim*_), so it keeps the threshold state until *t*_2_. **(B)** At *t* = *t*_0_, the future time *t*_*lim*_ is calculated using the value in the 32 − *bit* global counter. Only 8 bits of *t*_*lim*_ are stored ($t_{lim}^{8b}$), given by *b*_*TR*_. **(C)** The pixel reaches the threshold again at *t* = *t*_1_. As *t*_1_ < *t*_*lim*_, no output event should be allowed. For that, $t_{1}^{8b}$ and $t_{lim}^{8b}$ are compared.

Figure 6A illustrates how the neuron state is increased every time a new input event arrives until it reaches the threshold. At *t* = *t*_0_, an output event is generated and the neuron state is reset, so the controller block reads the present time *t*_0_ in the 32−*bit* global counter (as shown in Figure 6B) and calculates the future time when it will be allowed to generate an output spike again *t*_*lim*_. This future time is given by *t*_*lim*_ = *t*_0_ + *T*_*R*_ − Δ*t*, where *t*_0_ is the present time, *T*_*R*_ the minimum rate period or refractory time, and Δ*t* is a small correction applied to the calculations to compensate for frequency deviations. The calculation of this Δ*t* is described at the end of this section, so for now we will assume Δ*t* = 0. At *t* = *t*_1_, the neuron reaches the threshold (see Figure 6A), but it is not allowed to generate an output event, as *t*_1_ < *t*_*lim*_. Therefore, the neuron keeps the threshold value until *t*_2_, when a new input event is received and an output event is finally generated (because *t*_2_ > *t*_*lim*_). Although this output event should be generated ideally at *t*_*lim*_, we propose a computational simplification where the output event is not sent out until the pixel receives another input event at *t*_2_. The error Δ*t* introduced by this simplification is compensated in the calculation of the next *t*_*lim*_. A flag *f*_Δ*t*_ is used to indicate whether this compensation has to be applied or not.

The resolution of the global counter is 32 bits, so that is also the size of *t*_*lim*_. The rate saturation period memory stores information about *t*_*lim*_ for each pixel in a register, resulting a size of *n*_*x*_ × *n*_*y*_ × *n*_*bits*__*T*__*R*__ for this memory. If the whole *t*_*lim*_ is stored for each pixel, each memory register needs 32 bits and the memory consumes a lot of resources. We propose to reduce the size of this memory by a factor of 4, storing only 8 bits per pixel ($t_{lim}^{8b}$ in Figure 6B), from *b*_*TR*_ to *b*_*TR*_ − 7. The value of *b*_*TR*_ is a parameter that must be specified before implementation (7 ≤ *b*_*TR*_ ≤ 31), and it corresponds to the MSB (Most Significant Bit) of the rate saturation period *T*_*R*_. Therefore, the possible values of *T*_*R*_ that can be programmed after implementation will be given by $T_{R}^{min}=2^{b_{TR}-7}$ and $T_{R}^{max}=2^{b_{TR}+1}-1$.

**Figure 7:**
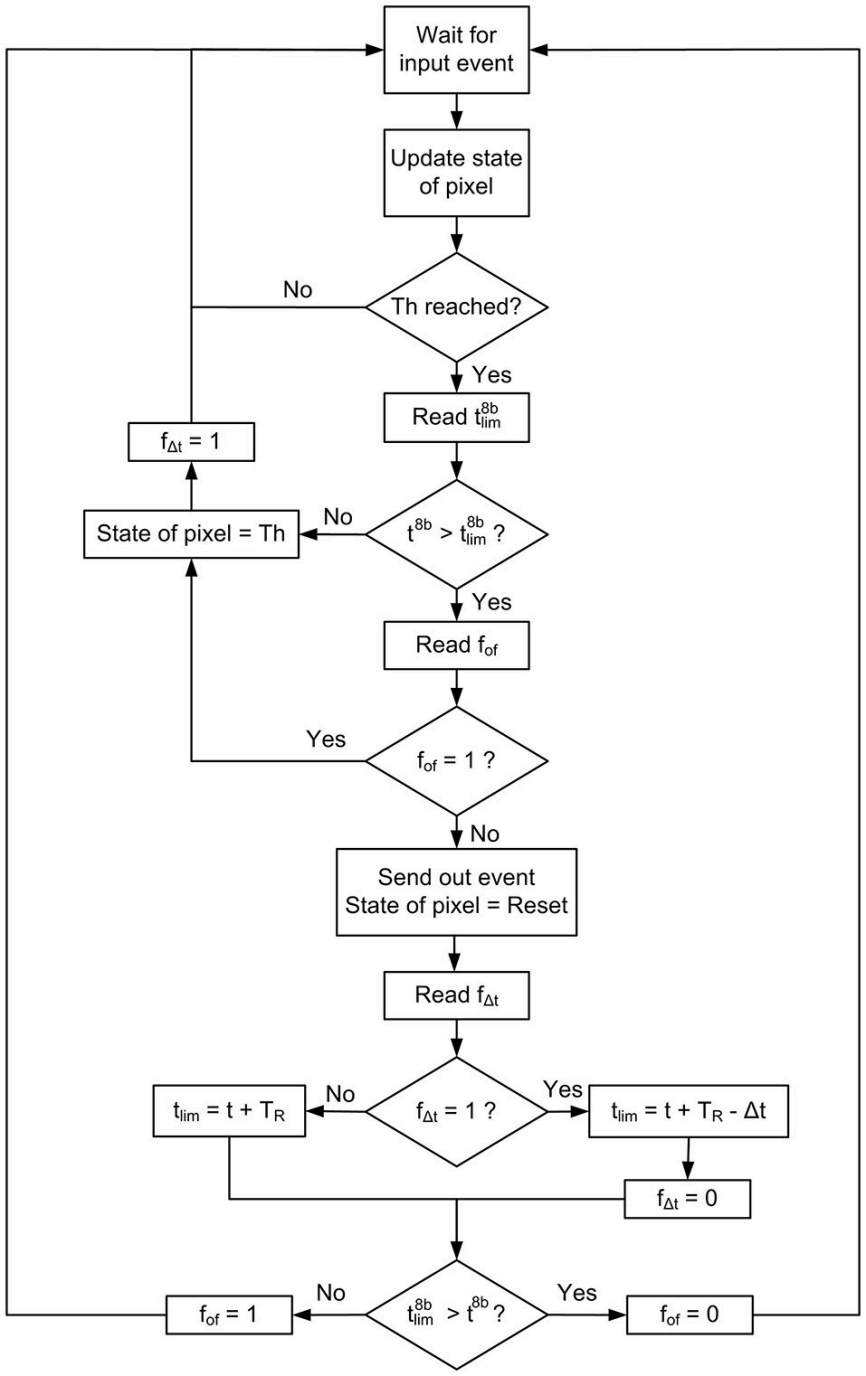
Flow diagram describing the rate saturation period mechanism.

According to this strategy, 8 bits from *t*_*lim*_ ($t_{lim}^{8b}$) are stored at the register of the rate saturation period memory corresponding to that specific pixel. After that, the next time this pixel reaches the threshold, the controller block reads the time *t*_1_ in the 32−*bit* global counter, extracts 8 bits from it ($t_{1}^{8b}$, from *b*_*TR*_ to *b*_*TR*_ − 7, as shown in Figure 6C) and compares it with $t_{lim}^{8b}$. If $t_{1}^{8b}>t_{lim}^{8b}$, it sends out the event; otherwise, it stores the threshold value in the corresponding position of the neuron memory and waits for the next incoming event. However, this mechanism can cause wrong decisions, as the bits more significant than *b*_*TR*_ are not compared (it can happen that $t_{1}^{8b}<t_{lim}^{8b}$ while *t*_1_ > *t*_*lim*_ due to overflow, so an output event would be missed). In order to avoid this kind of errors, a flag *f*_*of*_ indicating overflow is used together with a refresh mechanism. The use of this flag is described in Figure 7. Every time the threshold is reached after updating the state of a pixel with a new input event, the present value of the global counter (*t* in Figure 7) is used to find out if it is allowed to send out an event or not. For that, 8 bits from this counter (*t*^8*b*^) are compared with the stored value of $t_{lim}^{8b}$. If $t^{8b}<t_{lim}^{8b}$, the pixel is still under the *T*_*R*_ limitation, so its state is set to the corresponding threshold value and the flag *f*_Δ*t*_ is set to 1, meaning that a Δ*t* correction will be necessary. On the other hand, if $t^{8b}>t_{lim}^{8b}$, the controller block cannot be sure that *T*_*R*_ time is really over until it checks the overflow flag *f*_*of*_. If *f*_*of*_ = 1, the pixel is still limited by *T*_*R*_, so the threshold value is stored in the pixel's state and *f*_Δ*t*_ is also set to 1. If *f*_*of*_ = 0, the overflow flag is not active, so the event is sent out while the pixel's state is reset. After that, the value of the next *t*_*lim*_ is calculated, taking into account the value of Δ*t* as the difference between the current time and the previous *t*_*lim*_ (as illustrated in Figure 6A) when the flag *f*_Δ*t*_ = 1, and resetting this flag. After the calculation of the new *t*_*lim*_, the 8 bits $t_{lim}^{8b}$ are stored in the rate saturation mechanism memory, and if overflow is detected during the calculation ($t_{lim}^{8b}<t^{8b}$), the corresponding flag *f*_*of*_ is set to 1.

**Figure 8:**
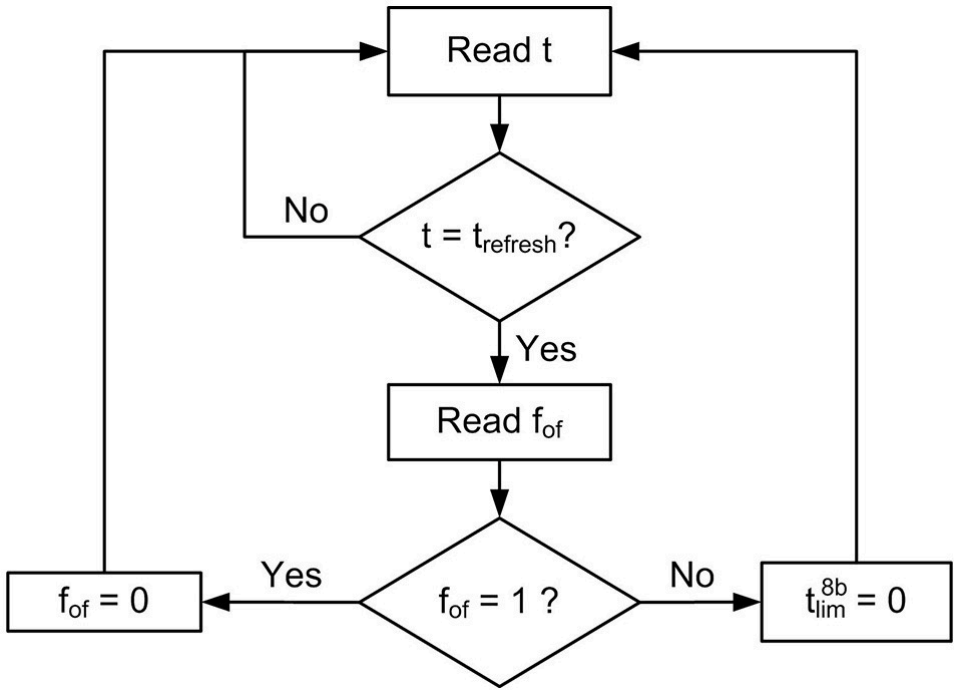
Flow diagram describing the refresh mechanism associated to the rate saturation period.

A global refresh mechanism is used to ensure the correct behavior of the overflow flag *f*_*of*_, as described in Figure 8. A refresh pulse is generated every time the global counter *t* reaches the value $t_{refresh}=2^{b_{TR}+1}-1$ (all bits from *b*_*TR*_ to *b*_0_ set to 1). When this happens, the controller block reads all *f*_*of*_ flags (one per pixel), and if *f*_*of*_ = 1, it is set to 0, indicating that overflow is not a problem anymore, while if *f*_*of*_ = 0, it resets $t_{lim}^{8b}=0$, indicating that *T*_*R*_ time is already over for that pixel.

Finally, the Δ*t* correction applied to the calculation of *t*_*lim*_ is also illustrated in Figure 6A. When the first output event is generated at *t*_0_, we assume Δ*t* = 0. However, the second output event occurs at *t*_2_, although it should have been generated at *t*_*lim*_. Therefore, the event generated at *t*_2_ had been delayed by the rate saturation mechanism, as indicated by the flag *f*_Δ*t*_ = 1 described before. In this case, the next *t*_*lim*_ is not measured from *t*_2_, but from the last *t*_*lim*_. In this particular example, after sending out the event at *t*_2_, the next *t*_*lim*_ would be calculated as *t*_*lim*_ = *t*_2_ + *T*_*R*_ − Δ*t*_2_. This correction is important to make sure that a neuron with rate saturation period *T*_*R*_ receiving an input train of events with frequency *f*_*in*_ higher than 1/*T*_*R*_ will generate an output train of events with average frequency given by *f*_*out*_ = 1/*T*_*R*_. Without this correction, *f*_*out*_ would be smaller, as it would depend of the exact arrival time of the input events. This Δ*t* does not introduce any error in the high temporal resolution of the events generated by a DVS, it actually introduces a correction in the effective value of the maximum event frequency given by the rate saturation period *T*_*R*_.

#### 2.1.4. Traffic control mechanism

As shown in Figure 3, the convolutional unit activates a *full*_*FIFO*_ signal when the output FIFO is full, and this signal is used to implement a traffic control mechanism at the network level. Considering that the network receives a flow of events from a DVS using AER protocol, we implemented a mechanism which drops input events whenever a node in the network has the *full*_*FIFO*_ signal active. Instead of holding the acknowledge and introducing artificial delays in the events flow, this mechanism reduces dynamically the amount of input events while keeping the spatio-temporal correlation between them. When the input event rate is too high, the network subsamples the input event flow, reducing the event rate as certain events are dropped. However, the precise timing of the events which are actually processed by the network is not altered. It is evident that the information carried by the dropped events is lost, but the information carried by the processed events is not modified by the network traffic, so the spatio-temporal correlation between them is kept.

The implemented traffic control mechanism introduces some uncertainty in the network behavior, as event dropping depends on each individual propagation delay inside the network, which is not completely deterministic. This effect can cause slightly different results when processing the same stimulus twice. We compensate this by analyzing statistically the behavior of the network after repeating each experiment up to 100 times, as shown in **Figure 14**.

### 2.2. Convolutional neural network for recognition tasks

**Figure 9:**
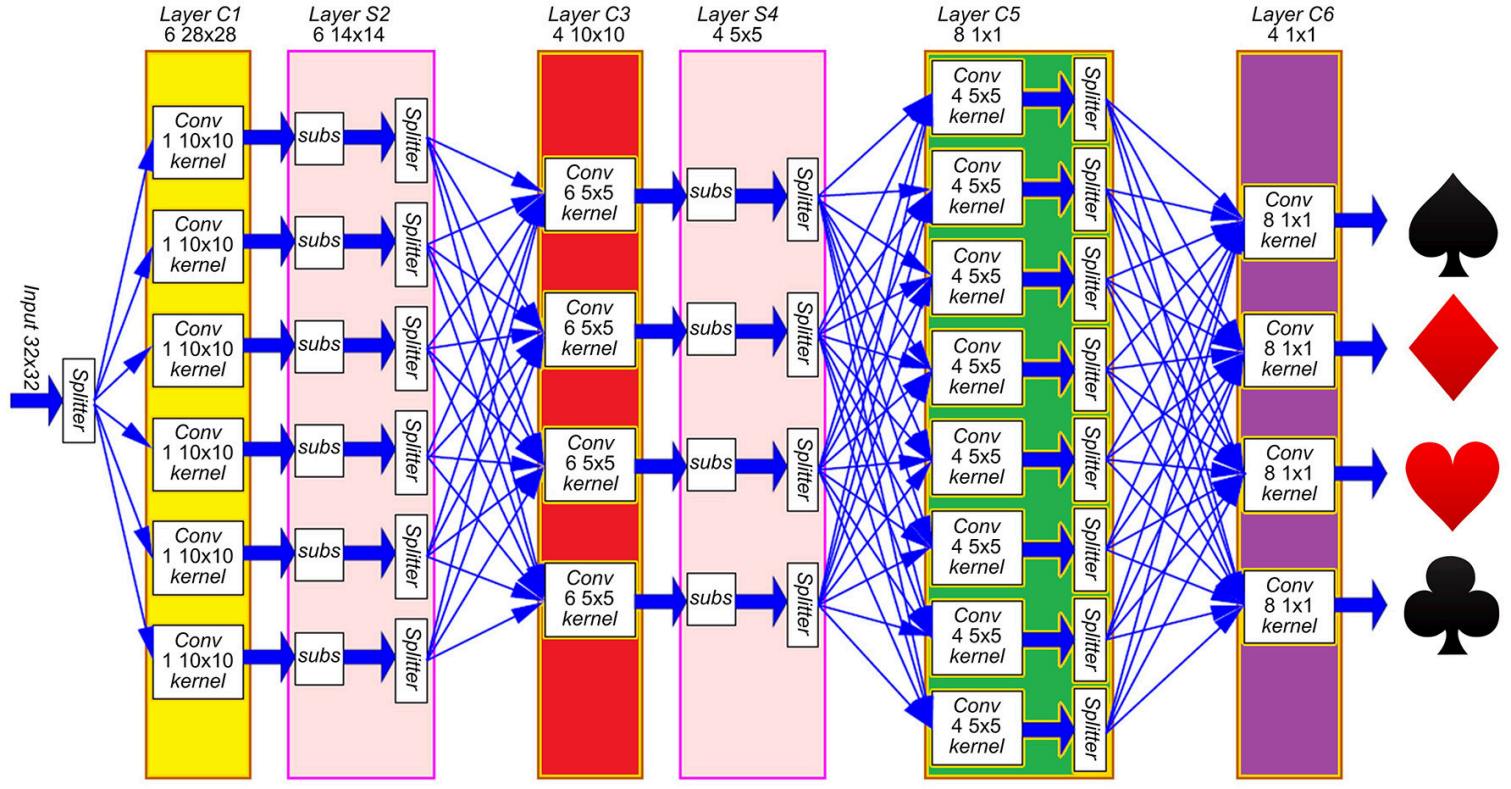
Schematic block diagram of the Convolutional Neural Network used for poker card symbol recognition. Yellow, red, green and purple boxes represent convolutional layers, while pink boxes correspond to subsampling layers. Layer C1 extracts oriented edges, which are downsampled by S2. Those different edges are combined by C3 and downsampled again by S4. Layer C5 obtains specific features that are combined by C6 to decide which of the four poker symbols is being observed.

**Figure 10:**
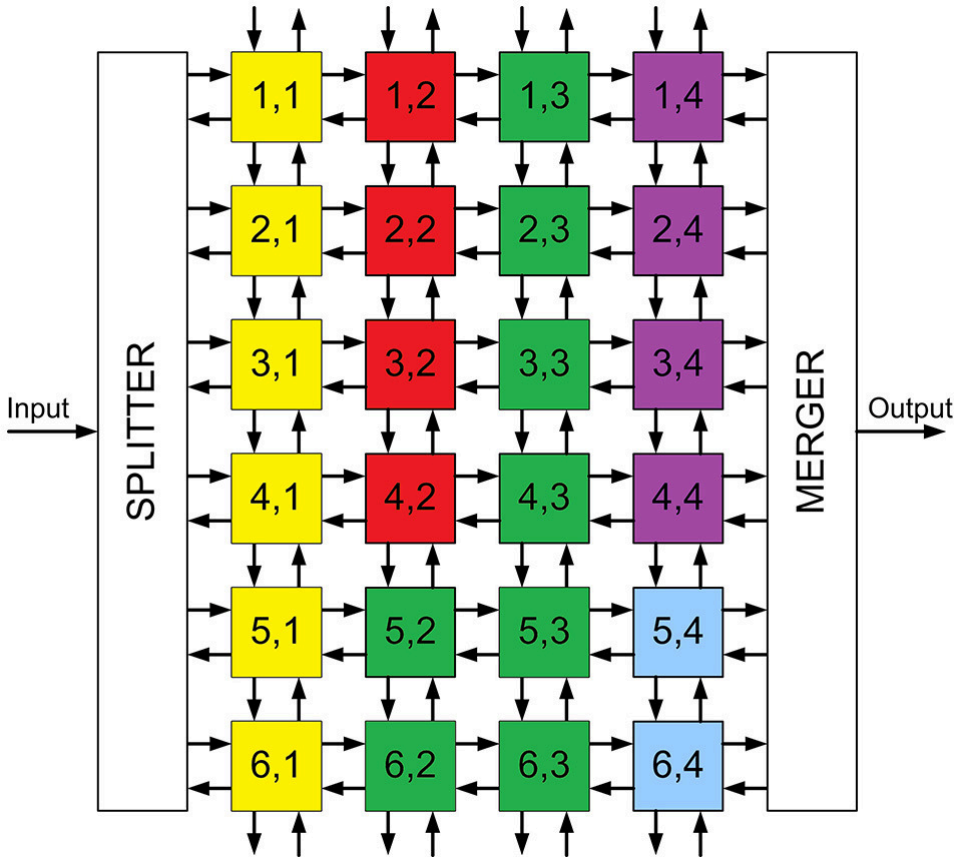
Schematic block diagram of the FPGA implementation of the Convolutional Neural Network used for poker card symbol recognition. Yellow modules represent layer C1, red modules correspond to layer C3, green modules correspond to layer C5 and purple ones to layer C6. Blue modules only include routing capabilities.

As described in Section 2.1, the convolutional node has been designed to assemble large 2D arrays in order to implement event-driven Convolutional Neural Networks (ConvNets). As an example, we implemented on FPGA the ConvNet described by (Pérez-Carrasco et al., 2013) for high-speed poker symbol recognition. The network is represented in Figure 9, and it consists of 4 convolutional layers (named C1, C3, C5 and C6 in the figure) and 2 subsampling layers (named S2 and S4). This figure shows that 22 convolutional modules are used to implement the whole network: 6 modules with 28 × 28 pixels in layer C1, 4 modules with 10 × 10 pixels in layer C3, 8 modules with 1 × 1 pixel in layer C5 and 4 modules with 1 × 1 pixel in layer C6. Subsampling layers S2 and S4 reduce the address space by a factor 2, from 28 × 28 to 14 × 14 and from 10 × 10 to 5 × 5, respectively. This is done by summing the events in each subsampling window of 2 × 2 pixels into a single pixel. A schematic block diagram of the hardware implementation on FPGA is represented in Figure 10, where all the modules are placed in an array of 6 × 4. The input splitter sends the incoming events to all 6 blocks in column 1 (*i*, 1)_*i* = 1, …, 6_, while the output merger receives all events from modules in column 4 (*i*, 4)_*i* = 1, …, 6_ and sends them out. The internal routers in each module are programmed to reproduce the connectivity of the network in Figure 9. Therefore, the yellow modules in Figure 10 correspond to layer C1, the red modules correspond to layer C3, the green blocks correspond to layer C5, and the purple ones to layer C6. The two blue modules only include the router for communication purpose, as no more convolutional modules are needed. Subsampling layers S2 and S4 are implemented by shifting the bits in the parallel buses between C1-C3 and between C3-C5, ignoring the Least Significant Bit in both x- and y-coordinates. Therefore, all events in each 2 × 2 subsampling window are summed into a single pixel.

The aim of this work is the implementation of a given ConvNet in a commercial FPGA, so we consider that the network has already been trained. In the particular example of ConvNet we use to illustrate our architecture, the network was implemented and trained in software by Pérez-Carrasco et al. (2013) in the frame domain using backpropagation, mapping the obtained parameters to the equivalent event-driven representation. Taking these parameters as a starting point, we have mapped them to our specific implementation.

The mapping of the network parameters from the values given by Pérez-Carrasco et al. (2013) (see row 1 in Table 1) can be described as a two-stage procedure. First, the amplitude parameters (kernel weights and convolution thresholds) and the time parameters (rate saturation periods and leakage rates) had to be adapted (scaled and rounded) to the hardware implementation, and second, they had to be tuned to compensate for the nonidealities of the hardware by using an optimization algorithm. The original amplitude values were represented in software using double-precision floating point numbers, while the proposed hardware implementation uses 9-bit integers for the neuron states (from 0 to 511). However, we compute negative values by shifting reset state to *Th*, and using 0 and 2 × *Th* as negative and positive thresholds, respectively. Although in principle it would be possible to use a maximum value of *Th* = 256, in practice it could cause overflow errors resulting in numbers larger than 511. We avoid this problem by setting a maximum value of *Th* = 128. Therefore, the first stage scales up all thresholds to 128, while keeping the corresponding kernel weights proportional for each layer. This change does not affect the rate saturation period values, but it does affect the leakage rate values as they are defined in Table 1. *LR*_*i*_ is defined as the ratio between the threshold value and the time it would take to decrease it until the reset value for layer *i*. Therefore, it also has to be scaled up with the threshold. The obtained parameters are shown in row 2 of Table 1. This first stage of the mapping is done automatically by a routine which reads all the indicated network parameters, and scales and rounds their values. However, the direct adaptation of these parameters does not produce the same behavior in the network, as the hardware implementation has some nonidealities that were not present in the software version. First of all, the network parameters have a smaller precision on hardware. Additionally, there will be some uncertainties in the hardware when two or more events should arrive at a single node ideally at the same time. In those cases, it is impossible to predict the order of processing the events, so that will affect the result of the convolutions. Finally, we implemented a traffic control mechanism in the network using the *full*_*FIFO*_ signal shown in Figure 3. This mechanism, as described in section 2.1.4, ignores the input events to the whole network every time one single module activates the *full*_*FIFO*_ signal, reducing the total amount of events processed by the network, but maintaining the spatio-temporal correlation among events. All these nonidealities change the behavior of the network, so we used simulated annealing to optimize this set of parameters. Each iteration sends the new values to the FPGA and evaluates the network, finally obtaining the optimized values shown in row 3 in Table 1.

**Table 1:** Network Parameters.

**Version**	***T*_*R*3_ (*ms*)**	***T*_*R*5_ (*ms*)**	***th*_1_**	***th*_3_**	***th*_5_**	***th*_5_**	***LR*_1_ (*s*^−1^)**	***LR*_3_ (*s*^−1^)**	***LR*_5_ (*s*^−1^)**	***LR*_6_ (*s*^−1^)**
Pérez-Carrasco et al. (2013)	0.10	0.46	0.64	1.42	7.36	2.17	0.72	0.90	1.21	0.72
FPGA scaled	0.10	0.46	128	128	128	128	143.62	81.16	21.00	42.41
FPGA optimized	0.08	0.43	66	114	93	126	3,959.93	3,576.70	906.61	214.88

In short, the mapping from the original network parameters takes two stages. In the first one, a routine scales and rounds the parameters automatically in a few seconds, while the second one fine-tunes the parameters doing simulated annealing and is much more time-consuming. The simulated annealing algorithm was run in a PC, which for each iteration sends the parameter values to the FPGA, sends a certain stimulus, records the output events and evaluates the performance. The stimulus used was part of a sequence of events obtained from a DVS (Serrano-Gotarredona and Linares-Barranco, 2013) which was observing a deck of 40 poker cards running in 1 second. In particular, we used the events corresponding to the initial 20 symbols with a slow-down factor of 10. The event rate of the real-time sequence was too high, so the number of dropped events would not give a good optimization of parameters. The simulated annealing algorithm needed 7421 iterations, which consumed around 10 h (the input stimulus takes 5 s). The whole stimulus with 40 symbols was used later on to characterize the network, as described in detail in Section 3.2. By using this DVS data, the network is trained to recognize specific temporal characteristics, so the temporal parameters of the network (rate saturation periods and leakage rates) are adapted to the speed of the training stimulus. If the stimulus is accelerated or decelerated, the temporal parameters have to be scaled according to this acceleration or deceleration. Therefore, if the spatio-temporal correlation of the data is modified, the performance of the network is affected negatively.

The number of kernels used by this network, as shown in Figure 9, is 94: 6 kernels (one per convolutional module) with 10 × 10 weights each in layer C1, 24 kernels (6 per convolutional module) with 5 × 5 weights each in layer C3, 32 kernels (4 per convolutional module) with 5 × 5 weights each in layer C5, and 32 kernels (8 per convolutional module) with 1 single weight each in layer C6. All kernel values were scaled and rounded versions of those trained by (Pérez-Carrasco et al., 2013), where the first layer corresponds to Gabor filters with different orientations to extract edges, while the other layers are the result of training with backpropagation and their shapes have no geometrical meaning.

## 3. Results

**Figure 11:**
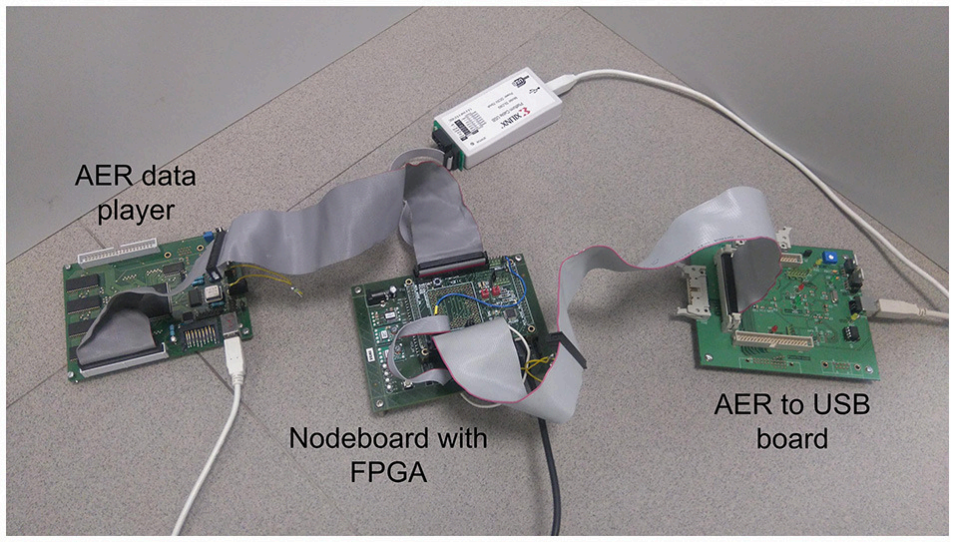
Photograph of the experimental setup used to characterize the convolutional network.

The experimental setup used to characterize both the isolated convolutional node and the whole network is shown in Figure 11. An AER data player board (Serrano-Gotarredona et al., 2009) receives a list of AER events through a USB port and sends the events out to the AER-node board (Iakymchuk et al., 2014), where a Spartan6 FPGA is used to implement the different processing systems. The AER-node board sends out events to another board which communicates with a PC through a USB port (Serrano-Gotarredona et al., 2009). A micro-controller in the AER-node board also receives the configuration parameters from a PC using a USB port and sends them to the FPGA through an SPI interface.

### 3.1. Characterization of convolutional node

**Figure 12:**
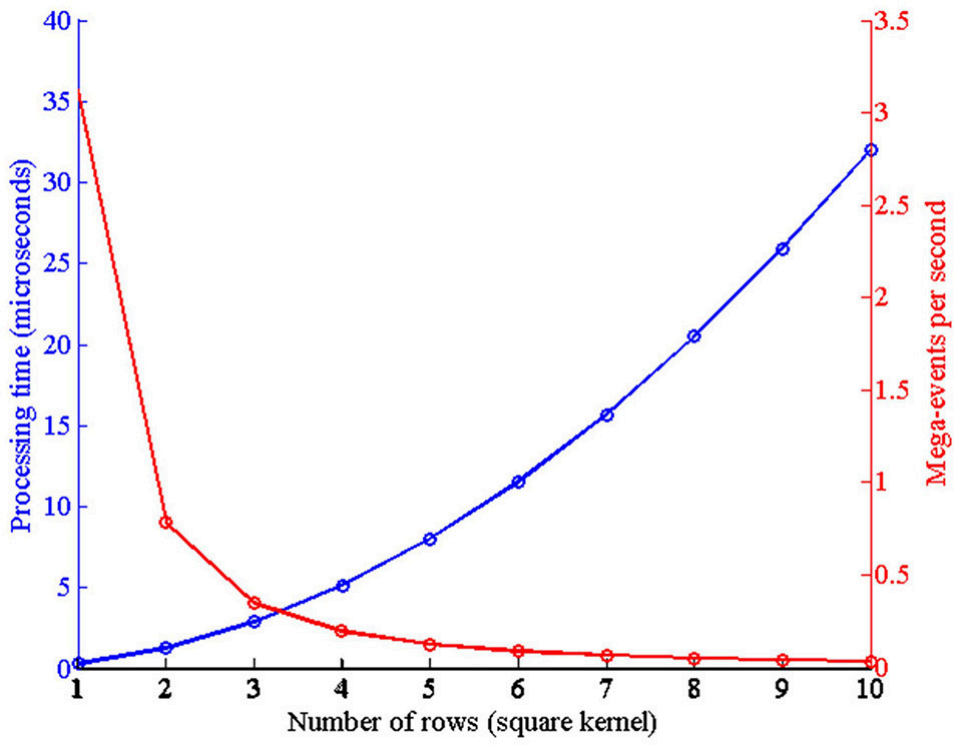
Left axis: Time spent by the convolutional module to process one event for different square kernel sizes, with *T*_*clk*_ = 20 ns. Right axis: Equivalent processing capabilities in Meps.

For the initial tests, a single convolutional node was implemented on the FPGA, like the one in Figure 2. The latency is measured as the time necessary to receive an input event, process it, and generate an output event, and it is given by the expression *T*_*event*_ = *T*_*routerin*_ + *T*_*ini*_ + *T*_*proc*_ + *T*_*routerout*_. When an incoming event is received from another node, first the router compares the event header with the local router address, and if both are the same it sends the event to its local convolutional unit (*T*_*routerin*_ = 6 clk cycles). After the event is received by the local processor, it compares the event address with the kernel parameters, and it calculates all the operations that have to be done to calculate the convolution (memory positions that have to be read for both pixel and kernel values, portions of the kernel which fall outside of the visual space of the module). We call this time *T*_*ini*_, and it consumes 37 clk cycles. *T*_*proc*_ is the time spent doing the convolution itself, and it is proportional to the size of the kernel (16 × *size*_*kernel*_). Finally, assuming that after processing the whole kernel it generates an output event, the router receives this event from the local unit and sends it out to the next node (*T*_*routerout*_ = 4 clk cycles). Figure 12 represents the values measured for *T*_*proc*_ for different square kernel sizes, being our clock frequency *f*_*clk*_ = 50 MHz (equivalent *T*_*clk*_ = 20 ns). The red trace in the figure represents the equivalent computational capabilities in Meps (Mega-events per second).

**Figure 13:**
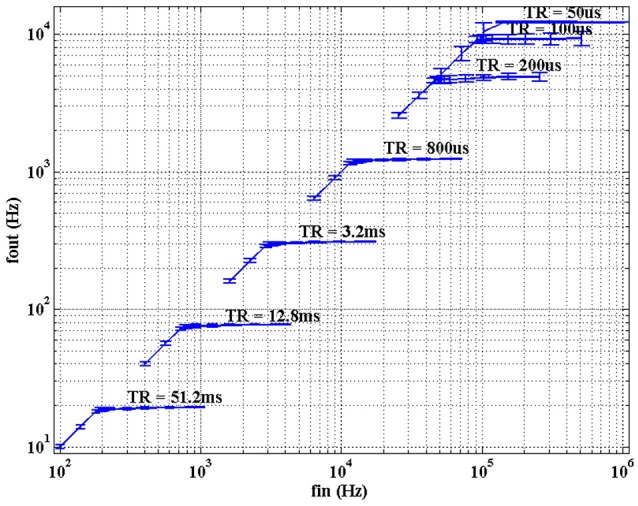
Characterization of the rate saturation mechanism for one single convolution pixel with *kernel* = 1 and *th* = 10. A train of input pulses with average frequency *f*_*in*_ and *std*_*in*_ = 10% is processed by the pixel, generating a train of output pulses with average frequency *f*_*out*_ and the *std* represented in the figure. Each trace corresponds to a different value of *T*_*R*_.

In order to characterize the rate saturation mechanism, different values of *T*_*R*_ were programmed covering the whole desired working range (51.2 ms, 12.8 ms, 3.2 ms, 800μs, 200μs, 100μs, 50μs). For each case, a 1 × 1 kernel with value 1 and a threshold *Th* = 10 were configured. The input stimulus is a train of events with fixed address and inter-spike interval following a normal distribution with mean 1/*f*_*in*_ and standard deviation *std*_*in*_ = 10% of the mean. Therefore, if there was no refractory limitation, the average frequency of the output train of events would be *f*_*out*_ = *f*_*in*_/10, with the same standard deviation. Figure 13 shows *f*_*out*_ vs. *f*_*in*_, where each trace corresponds to a different value of *T*_*R*_. The error bars represent the standard deviation of the measured output frequencies. Having a closer look at the segment for *T*_*R*_ = 51.2 ms, there is a saturation frequency *f*_*sat*_ = 1/*T*_*R*_ = 19.53 Hz, so for values of *f*_*in*_ < *Th* × *f*_*sat*_ = 195.3 Hz there is a linear relationship, while larger input frequencies produce saturation.

As *T*_*R*_ decreases in Figure 13, the different traces reproduce the same behavior, until the inter-spike interval becomes comparable to the global refresh pulse applied by the rate saturation mechanism.

### 3.2. Network characterization

The convolutional neural network described in Section 2.2 was implemented on the FPGA and tested using the experimental setup shown in Figure 11. This network consists of 22 convolutional nodes distributed in 4 layers, with a total number of 5, 116 neurons and 531, 232 synapses, and consumed 93% of the available slices on the Spartan6 FPGA (21, 465 out of 23, 038). Table 2 indicates the FPGA utilization in terms of slices, registers and block RAMs for the whole network and four different nodes, one corresponding to each convolutional layer. Tables 3–6 show further details for the different blocks inside each convolutional node in layers C1, C3, C5, and C6, respectively.

**Table 2:** FPGA utilization for the whole network and convolutional nodes.

**Module**	**# of slices**	**# of registers**	**# of block RAMs**
Network	21,465	38,451	202
Node in C1	769	1,529	4
Node in C3	2,010	1,892	5
Node in C5	738	1,715	3
Node in C6	538	1,350	2

**Table 3:** Detailed FPGA utilization for a convolutional node in layer C1.

**Module**	**# of slices**	**# of registers**	**# of block RAMs**
Router	303	810	0
Convolutional unit	466	719	4
Address calculation block	195	107	0
Kernel memory	0	0	3
Neuron memory	0	0	1
SPI slave	16	55	0
FIFO in	19	29	0
FIFO out	11	16	0

**Table 4:** Detailed FPGA utilization for a convolutional node in layer C3.

**Module**	**# of slices**	**# of registers**	**# of block RAMs**
Router	379	963	0
Convolutional unit	1,631	929	5
Address calculation block	164	87	0
Kernel memory	0	0	3
Neuron memory	0	0	1
SPI slave	16	55	0
FIFO in	19	27	0
FIFO out	9	16	0

**Table 5:** Detailed FPGA utilization for a convolutional node in layer C5.

**Module**	**# of slices**	**# of registers**	**# of block RAMs**
Router	477	1,252	0
Convolutional unit	261	463	3
Address calculation block	44	25	0
Kernel memory	0	0	3
SPI slave	16	55	0
FIFO in	18	25	0
FIFO out	11	16	0

**Table 6:** Detailed FPGA utilization for a convolutional node in layer C6.

**Module**	**# of slices**	**# of registers**	**# of block RAMs**
Router	399	1,035	0
Convolutional unit	139	315	2
Kernel memory	0	0	2
SPI slave	14	55	0
FIFO in	15	18	0
FIFO out	10	16	0

**Figure 14:**
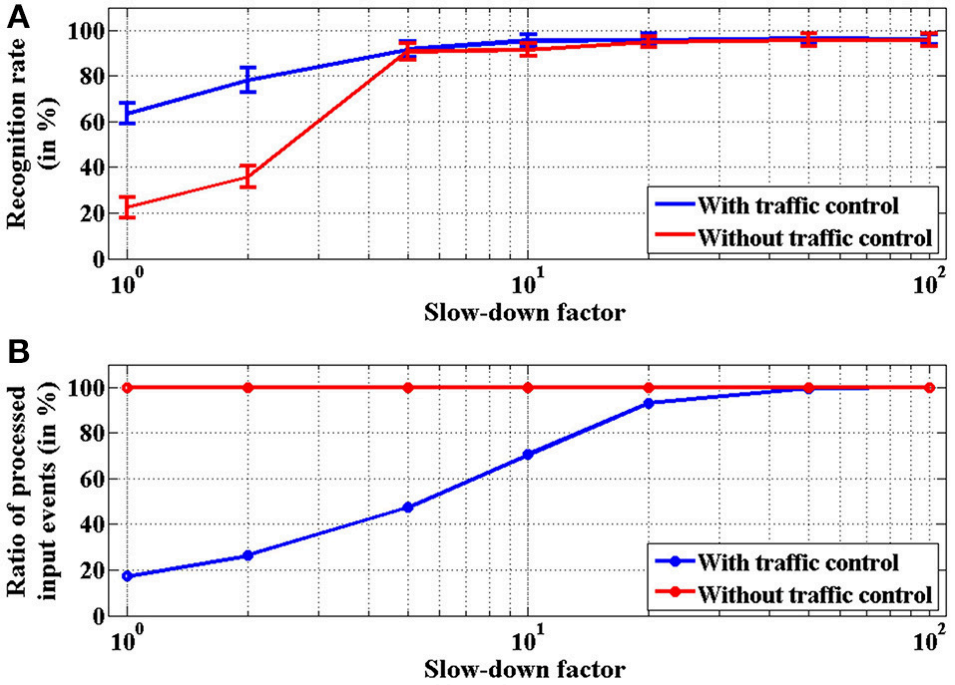
Characterization of the network for poker card symbol recognition with different values of the slow-down factor. **(A)** Measurement of the recognition rate (in %), representing the proportion of symbols identified correctly. The error bars were obtained by repeating each experiment 100 times. The blue trace corresponds to the proposed network including traffic control mechanism, while the red trace does not include that mechanism. **(B)** Proportion of input events (in %) processed by the network. When no traffic control is implemented (red trace), all input events are processed. Blue trace illustrates how the proposed traffic control mechanism implements temporal subsampling of events.

This network was adapted from the one proposed by Pérez-Carrasco et al. (2013) for poker card symbol recognition, following the procedure described in Section 2.2. In order to characterize this network, a sequence of events was reproduced by the AER data player and sent to the FPGA. These events were previously recorded using a Dynamic Vision Sensor (DVS) (Serrano-Gotarredona and Linares-Barranco, 2013) which was observing a deck of 40 poker cards running in roughly 1 s. The recorded events were pre-processed to track the symbols and extract a 32 × 32 pixels window of the whole visual field showing only the centered 40 symbols. This stimulus consists of 174, 644 events with an exact duration of 950 ms, which corresponds to an average event rate of 184 Keps (events per second). When this stimulus is processed by this convolutional neural network configured for symbol recognition, the total traffic registered inside the network is formed by 3, 172, 361 events, which corresponds to an event rate of 3.34 Meps. This event rate is higher than the capabilities of the network, specially limited by the event processing time in the first layer, where the size of the convolutional kernels is 10 × 10. From Figure 12, we can see that for a 10 × 10 kernel, one event requires a 32μs processing time. This limitation is overcome by the traffic control mechanism described in section 2.1.4. This mechanism discards input events whenever any convolutional block is in saturation (*full*_*FIFO*_ active), implementing a temporal subsampling of the input sequence, without altering the spatio-temporal correlation of the events within the system. In order to test the behavior of the network when processing this stimulus, different slow-down factors were applied to the play-back of the input events (100, 50, 20, 10, 5, 2, and 1) to reduce the rate of the input data. A slow-down factor of 1 indicates that the stimulus is played-back at real time, while a different value represents how many times slower it is played-back (2 times slower, and so on), scaling the precise timing of each individual event. For each slow-down factor, the time constants of the network were also scaled proportionally. Figure 14 shows the behavior of the network for poker card symbol recognition for each value of the slow-down factor, illustrating the effect of the proposed traffic control mechanism.

For each value of the slow-down factor, the events sequence of the 40 poker symbols was processed by the network 100 times in order to analyze its statistical behavior. For each time interval associated to one of the 40 symbols, the output events generated by the four neurons in the last layer were observed. Positive events indicate a symbol recognition, so we count the positive events generated by each of these output neurons, obtaining *n*_*s*_, *n*_*h*_, *n*_*d*_ and *n*_*c*_ (number of positive events generated by the output neurons associated to spades, hearts, diamonds and clubs, respectively). For example, if *n*_*s*_ > *n*_*h*_, *n*_*d*_, *n*_*c*_, we consider that the network recognized a spade. Following this criterion, we measured the recognition rate for each input trial as the number of symbols recognized correctly over the total number of symbols (which is 40 in our case). Figure 14A shows a comparison between the recognition rates obtained for the implemented network with the proposed traffic control mechanism (blue trace) and those obtained for the network without traffic control (red trace). For slow-down factors larger or equal than 5, the recognition rates obtained for both networks are almost identical (above 90%), while larger event rates (slow-down factors 1 or 2) demonstrate the advantages of the proposed method, with recognition rates around 65 and 22%, respectively, when processing the recording in real time. Figure 14B shows the proportion of input events actually processed in each case. When there is no traffic control, all events are processed by the network (although they are delayed by the handshake protocol between different convolutional blocks, altering the spatio-temporal correlation of the events), as represented by the red trace. However, the proposed mechanism discards input events when any convolutional block is saturated, producing a reduction of the number of processed events as the event rate increases, as shown by the blue trace. The robustness of the network is demonstrated by the fact that, even when only a small fraction of the input events is processed, the measured recognition rates are still reasonable. In the most conservative case (slow-down factor 100), the recognition rate is larger than 96%, with 100% of the input events actually processed. When the slow-down factor is 5, the proportion of processed events drops dramatically to around 45% while the recognition rate is still larger than 90%. Even when the recording is processed at real time, a recognition rate of around 65% is obtained with less than 20% of the input events. This example illustrates the robustness of this approach even when using a very slow clock signal (50 MHz) in an old FPGA, showing how a very small number of events is giving a reasonable high recognition rate. However, the capabilities of the proposed architecture to process high-speed stimuli in real time would increase dramatically using a modern FPGA with a faster clock. Another alternative to further reduce the processing time per second would be a VLSI implementation of this architecture, where a whole row of neurons could be updated in parallel as demonstrated in (Camuñas-Mesa et al., 2012).

**Figure 15:**
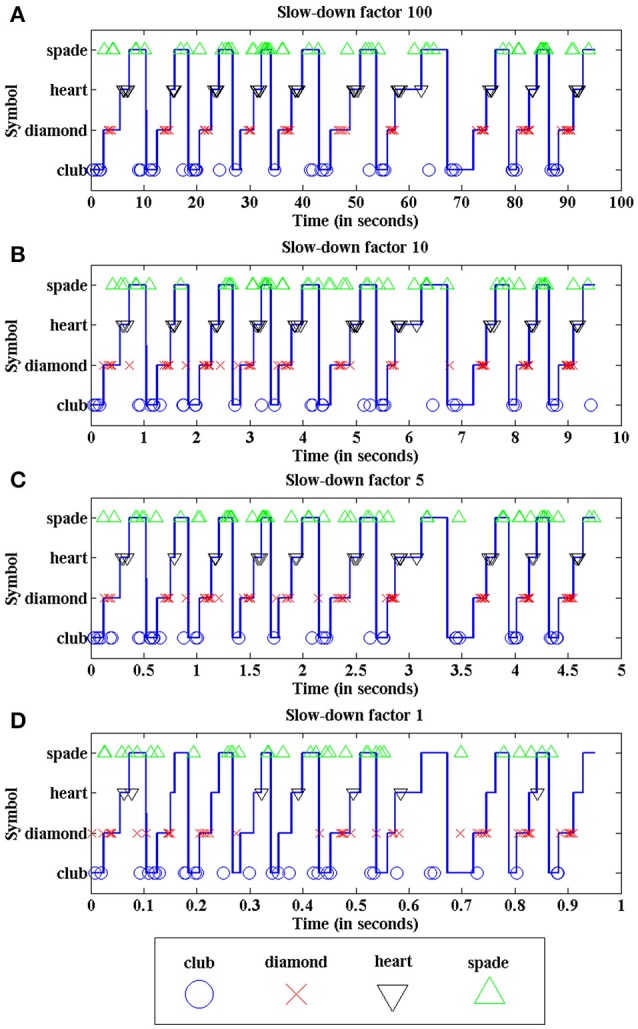
Recognition performance of the network for different slow-down factors: **(A)** 100, **(B)** 10, **(C)** 5, and **(D)** 1. Continuous blue traces represent the input symbol vs. time. Each individual marker represents a single positive event generated by a specific output neuron associated to spade, heart, diamond or club (see legend below).

Figure 15 illustrates the recognition performance for slow-down factors 100 (a), 10 (b), 5 (c), and 1 (d). In each plot, the continuous blue trace represents the input poker card symbol presented at each time, repeating the sequence club-diamond-heart-spade 10 times for each trial. The output events generated by the last layer of the network are represented by different markers for each output neuron associated with a symbol: blue circles for club, red crosses for diamond, black inverted triangles for heart, and green non-inverted triangles for spade. In Figure 15A, with slow-down factor 100, the best possible performance is shown, with a recognition rate of 97.5% (one symbol incorrectly classified out of 40). Despite this almost perfect performance, frequent false positives are shown in this plot, mostly between spade and club, as the lower part of both symbols is identical. Therefore, it is reasonable to assume that it might be difficult to distinguish between both symbols while they are moving and they are not completely visible all the time. However, the correct neuron always generates more positive events than the wrong ones (except in one case, in this particular example).

In Figure 15B,C, the proportion of processed input events decreases as the event rate increases, producing more false positives than in (a), as the shape of the symbols is less clear due to the sub-sampling of events. However, the recognition rates are still higher than 90%. Finally, Figure 15D shows the performance of the network when the input events are sent at real time, discarding more than 80% of them. In this case, a human observer looking at the visual information provided by the processed events (which represent less than 20% of the original event flow) would not be able to recognize easily the shapes of the symbols, as they are not complete. Even with these limitations, we obtain a recognition rate of 70% in this example, illustrating the robustness of the network.

**Figure 16:**
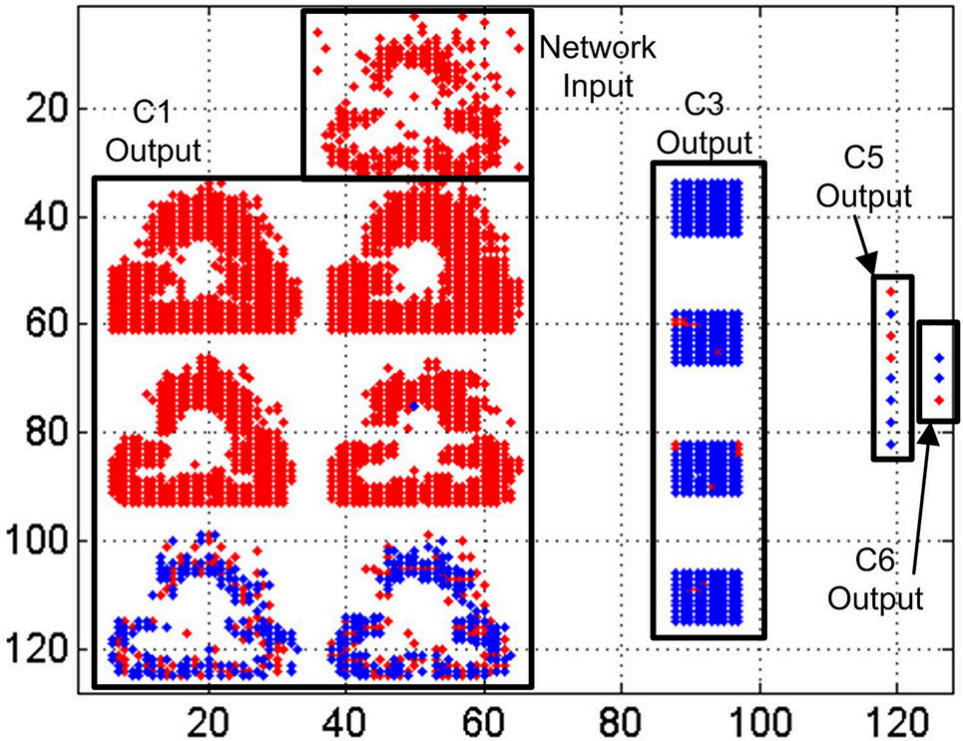
Reconstruction of the whole network activity for a slow-down factor of 100 during 100 ms. Red dots represent positive events, and blue dots represent negative events.

In Figure 16 we represent a reconstruction of the whole network activity for a slow-down factor of 100 during 100 ms. We plot in (*x, y*) space all the events generated by all the neurons during a 100 ms time period together with the simultaneous input events, with red dots representing positive events and blue dots representing negative events. The top black rectangle shows the network input during that period, which corresponds to a club symbol. Below that rectangle, we can see the output events generated by the 6 convolutional nodes in layer *C*1, with 28 × 28 neurons each. At the right hand side, another rectangle shows the output events generated by the 4 convolutional nodes in layer *C*3, with 10 × 10 neurons in each node. The next smaller rectangle shows the 8 convolutional nodes in layer *C*5, with one single neuron per node, showing either positive or negative activity. Finally, the smallest rectangle at the right side of the figure shows the output activity generated by layer *C*6, with 4 individual neurons, one for each symbol. In this case, the first neuron is not firing events during this time window, while the second and third neurons are firing negative events (blue dots) and the fourth one is firing positive events (red dot). This last neuron is the one attached to the club symbol, representing a correct recognition.

**Figure 17:**
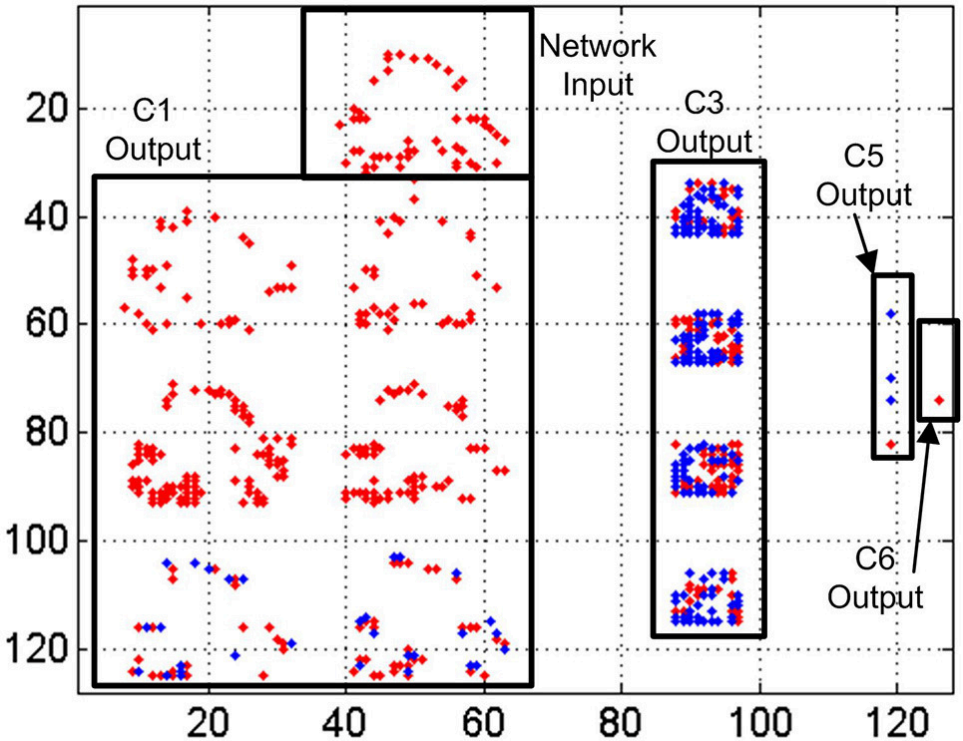
Reconstruction of the whole network activity for a slow-down factor of 1 during 1 ms. Red dots represent positive events, and blue dots represent negative events.

In Figure 17 we represent a reconstruction of the whole network activity when processing the input events at real time during 1 ms. As we mentioned before, only a small fraction of the input events is processed by the network at this high speed (less than 20% on average), which explains the higher sparsity of neuron activity in this figure. The top black rectangle again represents the network input during the 1 ms window. Theoretically, we should see the same activity than in Figure 16, as now we have increased the events speed by a factor of 100 while reducing the time window with the same factor. However, the traffic control mechanism implemented in the network discards more than 80% of the input events at this speed, almost ruining the shape of the input club, as shown in the top rectangle of Figure 17, which is barely recognizable by a human observer. However, the output of layer *C*6 shows positive activity at the correct neuron, while no activity at all is observed for the other ones. Although we can see very sparse activity at all the layers of the network, that activity is enough to obtain a correct symbol recognition, demonstrating the robustness of the network.

**Figure 18:**
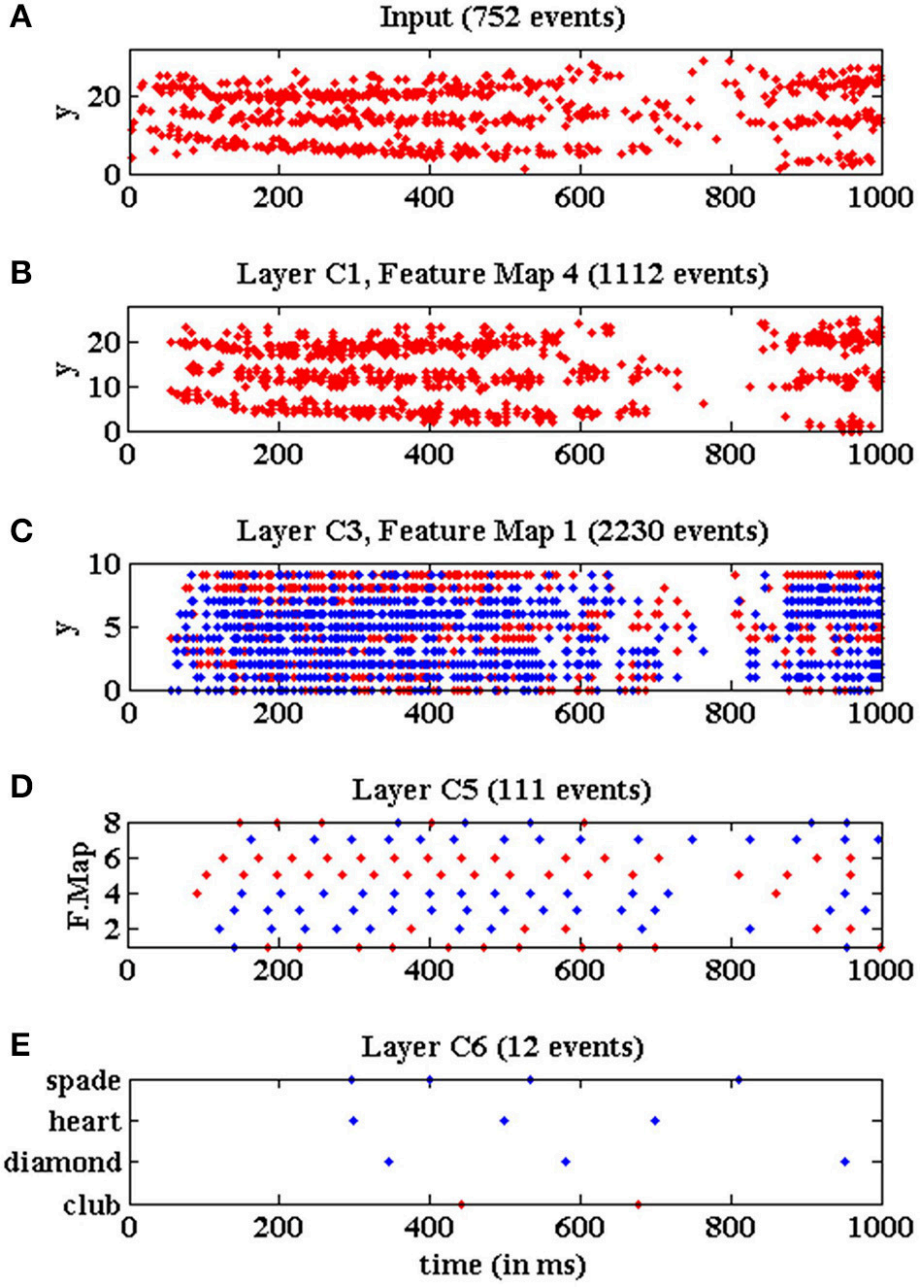
Events vs. time representing the network activity for a slow-down factor of 100 during a time window of 1 s, when processing a club symbol. Red dots represent positive events, and blue dots represent negative events. **(A)** Y-coordinates vs. time of the input events generated by the DVS and processed by the network while a club symbol is moving for 1 s. **(B)** Y-coordinates vs. time of the output events generated by convolutional node number 4 in layer *C*1. **(C)** Y-coordinates vs. time of the output events generated by convolutional node number 1 in layer *C*3. **(D)** Convolutional node number vs. time of the output events generated by layer *C*5. **(E)** Neuron label vs. time for the output events generated by layer *C*6.

The event timing of the multi-layer network is illustrated in Figure 18, where we plot events vs. time during 1 s while a club symbol is being processed with a slow-down factor of 100 (red circles represent positive events, and blue crosses represent negative events). This time window of 1 s represents only an initial cut of the whole symbol sequence, which lasts around 2.5 s in this example. Figure 18A shows the y-coordinates of the flow of events generated by the DVS sensor while the club symbol is moving in front of it. At this time window, 752 events were received by the network, while the whole symbol sequence consists of a total number of 5, 583 events (an average input event rate of 2.23 Keps for this symbol). Figure 18B shows the y-coordinates of the output events generated by the fourth convolutional node in layer *C*1. This plot illustrates the pseudo-simultaneity of event-based processing (Farabet et al., 2012), as this first layer is producing output events which are simultaneous with the input ones, introducing only an initial latency between input and output sequences. This node generated 1, 112 events during the represented time window, while a total number of 9, 449 events were produced during the processing of the whole symbol. Figure 18C shows the y-coordinates of the events generated by the first convolutional node in layer *C*3. This node generated 2,230 events during the initial cut, with a total of 11, 308 events for the whole sequence. Figure 18D shows the activity generated by layer *C*5. As each convolutional node in this layer consists of a single neuron, the whole layer can be represented by showing the events generated by the 8 nodes. These 8 nodes produced 111 events during the initial time window, while 349 events were generated during the whole symbol processing. Finally, Figure 18E shows the activity generated by layer *C*6, which is formed by 4 neurons, each one associated to a different poker symbol. In this example, the neuron associated to the club symbol generates two positive events, while the other neurons generate several negative events, concluding that the symbol was recognized correctly. The latency between the beginning of the stimulus and the first recognition event is less than 450 ms for a slow factor of 100. This last layer generated 12 events during the initial window of 1 s, while the complete sequence produced a total number of 35 events.

**Figure 19:**
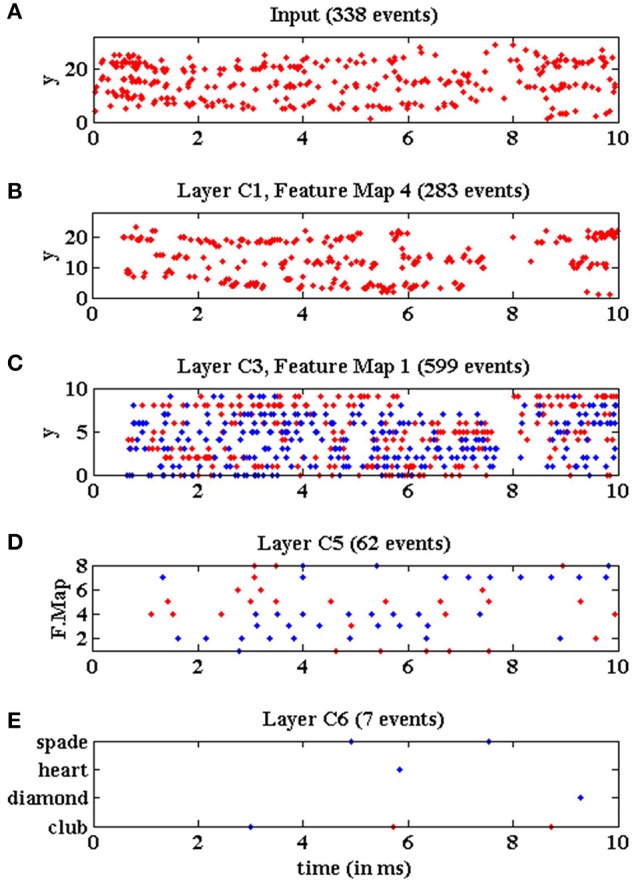
Events vs. time representing the network activity at real time during a time window of 10 ms, when processing a club symbol. Red dots represent positive events, and blue dots represent negative events. **(A)** Y-coordinates vs. time of the input events generated by the DVS and processed by the network while a club symbol is moving for 10 ms. **(B)** Y-coordinates vs. time of the output events generated by convolutional node number 4 in layer *C*1. **(C)** Y-coordinates vs. time of the output events generated by convolutional node number 1 in layer *C*3. **(D)** Convolutional node number vs. time of the output events generated by layer *C*5. **(E)** Neuron label vs. time for the output events generated by layer *C*6.

Figure 19 illustrates the behavior of the network when processing the same input stimulus at real time. In this case, the 5, 583 events corresponding to this symbol should be processed in around 25 ms, giving an average input event rate of 223 Keps. In this figure, we plot the events recorded at the different layers of the network during an initial time window of 10 ms while processing the same club symbol. Figure 19A shows the y-coordinates of the events generated by the DVS sensor. For this event rate, the traffic control mechanism implemented in the network limited the total amount of events that could be processed by the system, ignoring the input events while the *full*_*FIFO*_ signal became active in any convolutional node. For this reason, only 338 input events are shown in Figure 19A, representing around 45% of the events shown in Figure 18A for the initial time window. However, if we plotted the whole symbol sequence, we would see that the total number of input events processed by the network is 936, representing less than 20% of the whole sequence, which is consistent with Figure 14B for slow-down factor 1. Figure 19B shows the y-coordinates of the events generated by the fourth convolutional node in layer *C*1, only 283 events at this time window from a total number of 874 events for the whole 25 ms sequence. Figure 19C shows the y-coordinates of the events generated by the first convolutional node in layer *C*3, with 599 events presented in the figure from a total number of 1, 260 for the whole symbol processing. Figure 19D shows the activity generated by all the convolutional nodes in layer *C*5, which corresponds to 62 events during the 10 ms time window, and a total of 137 for the whole sequence. Finally, Figure 19E shows the activity generated by the 4 output neurons in layer *C*6, with only 7 events in the presented cut, and a total number of 15 events for the complete symbol processing. The figure shows how the neuron associated to the club symbol generates two positive events and a negative one, while the other neurons generate only negative events, so the symbol was recognized correctly. The latency between the onset of the stimulus and the first positive event is less than 6 ms at real time, which represents a very fast recognition task. Although the proportion of events processed by the network is less than 20% at real time, this example illustrates how a successful recognition is obtained by exploiting the robustness of the network.

The power consumed by the whole network inside the FPGA was measured while processing the input sequence for different slow-down factors, obtaining 7.7 mW when the stimulus was being processed at real time, and even lower consumptions for slower processing: 5.25 mW when it was 10 times slower, and 0.85 mW for a slow-down factor of 100. Considering that each symbol was presented for an average time of 25 ms in the real-time situation, the energy per classification can be obtained as 192.5μJ, or inversely a number of 5, 194.81 classifications per Joule. The same calculations can be done for slow-down factor of 10 with *T*_*symbol*_ = 250 ms, and for a slow-down factor of 100 with *T*_*symbol*_ = 2.5 s. Table 7 summarizes the main results given in this section for different slow-down factors.

**Table 7:** Summarized main results.

**Measurement**	**Real-time**	**Slow-down factor 10**	**Slow-down factor 100**
Recognition rate	63%	95%	96%
Ratio of processed events	17%	70%	100%
Power consumption	7.7 mW	5.25 mW	0.85 mW
*T*_*symbol*_	25 ms	250 ms	2.5 s
Energy per classification	192.5 μ J	1.312 mJ	2.125 mJ
Classifications per Joule	5,194.81	761.90	470.58

## 4. Discussion

In recent years, the field of machine learning has experienced a huge progress taking advantage of the availability of immense image databases and the current computing power. Different tasks have attracted the attention of researches, like classifying handwritten digits from the MNIST dataset (LeCun and Cortes, 1998) or classifying an object into one of 1000 classes, as is required for the ImageNet dataset (Russakovsky et al., 2015). For instance, modern ConvNets like LeNet-5 (LeCun et al., 1998), AlexNet (Krizhevsky et al., 2012), GoogLeNet (Szegedy et al., 2015), VGG-16 (Simonyan and Zisserman, 2014) or ResNet (He et al., 2016) have been developed to solve such tasks with impressive results. However, the main bottlenecks in these applications are related to speed and power. The human brain represents a source of inspiration to design better approaches, given its ability to perform classification tasks in real time with a very small power consumption, so that is the goal of neuromorphic systems like the one presented in this work. While conventional frame-based ConvNets process huge amounts of data, neuromorphic systems process visual information encoded in events as generated by DVS sensors inspired by the biological retina, resulting in very sparse data which facilitates the reduction in processing time and power consumption. The pseudo-simultaneity property of neuromorphic systems allows individual events generated by the sensor to propagate through all the layers in the network immediately, while frame-based systems need to wait until large packages of information (frames) are processed by each layer, introducing multiple delays. Despite these clear advantages in neuromorphic systems, conventional frame-based ConvNets are still giving better performance, mostly due to the availability of image datasets mentioned before and very well-known training techniques based on frames, like backpropagation. Nevertheless, recent works have demonstrated similar performance in neuromorphic SNNs using event-based training techniques (Wu et al., 2017; Zheng and Mazumder, 2017), suggesting that it is only a matter of time that event-based ConvNets become competitive with respect to frame-based ones in terms of classification, while presenting better results in terms of speed and power consumption. Some frame-based approaches are using hardware accelerators (Aydonat et al., 2017; Qiao et al., 2017) to improve their performance in terms of speed. Some large-scale neuromorphic approaches (Schemmel et al., 2010; Benjamin et al., 2014; Furber et al., 2014; Merolla et al., 2014) have dealt with the speed/power tradeoff, showing very impressive results (Furber, 2016), although they are based on very expensive dedicated hardware. The architecture proposed in this paper allows for implementing large-scale ConvNets using cheap commercial FPGAs presenting competitive results in terms of the tradeoff recognition rate/speed/power.

A new configurable event-based convolutional node with rate saturation mechanism has been designed for hardware implementation of convolutional neural networks on FPGAs. This node was designed to assemble large 2D arrays, and includes three main blocks: (1) a processor unit, which calculates the convolutional operation of the input events with a programmable kernel and generates the corresponding output events, (2) a router, which manages the communication between the processor circuit and the neighboring modules, implementing the network structure, and (3) a configuration block, which receives commands through an SPI connection in order to set all the programmable parameters of the network. The proposed implementation of the rate saturation mechanism guarantees a programmable minimum separation in time between consecutive spikes for each single neuron, while the implemented traffic control mechanism discards input events when the network is busy, keeping spatio-temporal correlation and avoiding artificial delays. Although rectifying non-saturating non-linearities like ReLUs have been proposed as a simpler alternative to rate saturation mechanism in frame-based systems, they are not a good solution for spiking hardware implementation, as an excessively active neuron would generate a large amount of events and collapse the communication network. A Convolutional Neural Network with 4 layers and 22 nodes for poker card symbol recognition has been implemented on a Spartan6 FPGA using a 2D array of the proposed convolutional node. The individual node has been characterized for different rate saturation period values from 50μs to 51.2 ms, showing a correct behavior. The proposed network, with more than 5 K neurons and 500 K synapses, has been carefully characterized for the recognition of a sequence of 40 poker card symbols in 1 s time with different slow-down factors, from real time processing to 100 times slower. The slower versions showed recognition rates around 96% when all the input events were processed by the network, while less than 20% of the events were processed at real time, obtaining a recognition rate higher than 63%, demonstrating the robustness of the method even when the input stimulus is barely visible by a human observer due to the high speed. A recognition latency smaller than 6 ms was shown in the presented results. Arbitrary convolutional neural networks can be easily implemented using the proposed node and methodology, which can be expanded to multi-FPGA arrays by using appropriate I/O blocks reported elsewhere (Yousefzadeh et al., 2017). In the example presented in this paper, a relatively small FPGA was used with a slow clock signal (50 MHz). However, some newer FPGAs include more than 5 millions logic elements, and support maximum processing frequencies up to 1.5 GHz. This would imply around 36 times more slices and a clock signal 30 times faster. This number of slices would be able to fit up to 180 K neurons and 18 M synapses within a single FPGA.

## Author contributions

AL-B, TS-G, and BL-B conceived the idea and supervised the project. YD-C and LC-M developed the hardware implementation, and designed and conducted the experiments. LC-M wrote the paper with inputs from all the authors.

### Conflict of interest statement

The authors declare that the research was conducted in the absence of any commercial or financial relationships that could be construed as a potential conflict of interest.

## Footnotes

^1^Note that, if there is no non-linear saturation mechanism in a multi-layer ConvNet, so that all convolutional operations are linear, one could collapse all convolutions into a single one.
